# Wilms’ tumor 1-associating protein complex regulates alternative splicing and polyadenylation at potential G-quadruplex-forming splice site sequences

**DOI:** 10.1016/j.jbc.2021.101248

**Published:** 2021-09-25

**Authors:** Keiko Horiuchi, Takeshi Kawamura, Takao Hamakubo

**Affiliations:** 1 Department of Protein-Protein Interaction Research, Institute for Advanced Medical Sciences, Nippon Medical School, Tokyo, Japan; 2 Laboratory for Systems Biology and Medicine, Research Center for Advanced Science and Technology, The University of Tokyo, Tokyo, Japan

**Keywords:** WTAP, alternative splicing, polyadenylation, proteomics, G-quadruplex, cell cycle, AS, alternative splicing, DEG, differentially expressed gene, DEI, differentially expressed isoform, FMR1, fragile X mental retardation 1, FXR1, fragile X mental retardation syndrome-related protein 1, GO, gene ontology, HUVEC, human umbilical vein endothelial cell, KD, knockdown, m6A, N6-methyladenosine, PIR, percent intron retention, RIP, RNA coimmunoprecipitation, SR, serine-rich, WTAP, Wilms’ tumor 1-associating protein

## Abstract

Wilms’ tumor 1-associating protein (WTAP) is a core component of the N6-methyladenosine (m6A)-methyltransferase complex, along with VIRMA, CBLL1, ZC3H13 (KIAA0853), RBM15/15B, and METTL3/14, which generate m6A, a key RNA modification that affects various processes of RNA metabolism. WTAP also interacts with splicing factors; however, despite strong evidence suggesting a role of *Drosophila* WTAP homolog fl(2)d in alternative splicing (AS), its role in splicing regulation in mammalian cells remains elusive. Here we demonstrate using RNAi coupled with RNA-seq that WTAP, VIRMA, CBLL1, and ZC3H13 modulate AS, promoting exon skipping and intron retention in AS events that involve short introns/exons with higher GC content and introns with weaker polypyrimidine-tract and branch points. Further analysis of GC-rich sequences involved in AS events regulated by WTAP, together with minigene assay analysis, revealed potential G-quadruplex formation at splice sites where WTAP has an inhibitory effect. We also found that several AS events occur in the last exon of one isoform of *MSL1* and *WTAP*, leading to competition for polyadenylation. Proteomic analysis also suggested that WTAP/CBLL1 interaction promotes recruitment of the 3′-end processing complex. Taken together, our results indicate that the WTAP complex regulates AS and alternative polyadenylation *via* inhibitory mechanisms in GC-rich sequences.

Alternative splicing (AS) is the process by which a single gene produces multiple mRNAs, thereby substantially increasing both the protein diversity and complexity. Ninety-five percent of human multiexon genes are alternatively spliced, and the majority of these transcripts vary across tissues, developmental stage, or physiological state (1, 2). Splicing misregulation and the aberrant profile of AS are often associated with cancers and other human diseases (3, 4, 5). Recently, N6-methyladenosine (m6A), one of the most abundant internal mRNA modifications, which is catalyzed by the methyltransferase complex METTL3 and METTL14, has emerged as a key epitranscriptomic modification that affects RNA metabolism, including RNA splicing, mRNA stability, localization, and translation, which ultimately determine the expression of functional proteins (6, 7, 8, 9, 10, 11). Proteogenomics revealed that only 32% of the genes have statistically significant correlations between mRNA and protein abundance, suggesting the significance of RNA metabolism/processing (12). Recent studies have shown that m6A modification in mRNAs or noncoding RNAs influences multiple cellular processes in various organisms, including sex determination and dosage compensation in *Drosophila* and X chromosome inactivation in mammals, as well as viability, meiosis, tissue development, maternal-to-zygotic transition, stem cell self-renewal and differentiation, HIV replication, circadian clock control, DNA damage response, with additional links to obesity, cancer progression, and other diseases (13).

We have previously reported that Wilms’ tumor 1-associating protein (WTAP) regulates cyclin A2 mRNA stability and RNA splicing and is essential during mouse early embryo development and cell cycle progression in human umbilical vein endothelial cells (HUVECs) (14, 15, 16). Previous studies further revealed that WTAP is a regulatory subunit of the methyltransferase complex, which catalyzes the formation of N6-methyladenosine initially in *Arabidopsis thaliana* and yeast (17, 18). The interaction between WTAP and the methyltransferase catalytic subunits METTL3 and METTL14 has also been reported in various mammalian cells (15, 19, 20, 21). In addition, our previous proteomic study using HUVEC whole cell lysates showed that WTAP forms a protein complex comprising Virilizer homolog (VIRMA), CBLL1 (Hakai), ZC3H13(KIAA0853), which were identified as the major components isolated from all of the three different anti-WTAP antibodies with high total spectrum count in LC/MS/MS (15), and RBM15, as well as METTL3 and METTL14. All of them have been recently shown to participate in m6A methylation in various organisms (22, 23, 24, 25). Our proteomic study has also identified splicing factors including the arginine/serine-rich (SR) domain-containing proteins SRSF1, SRSF3, BCLAF1 and THRAP3, and hnRNPs, as well as the G-quadruplex binding proteins, fragile X mental retardation 1 (FMR1), Fragile X mental retardation syndrome-related protein 1 (FXR1) and FXR2 as WTAP-interacting proteins (15).

The *Drosophila* homologs of WTAP and VIRMA, Fl(2)d and Virilizer (Vir), respectively, are required for alternative splicing of *Sex lethal (SXL)*, a master regulator of *Drosophila* sex determination pathway (26, 27). The female-specific expression of the RNA-binding protein SXL regulates the AS and/or translation of *SXL*, *transformer(tra)*, and *male-specific-lethal 2 (msl-2)* pre-mRNA, which controls sex determination, sexual behavior, and dosage compensation. Recent studies have shown that this process is affected by m6A modifications, catalyzed by Mettl14 and Ime4 (the *Drosophila* homologs of METTL3) (28, 29). Moreover, biochemical analyses have revealed the physical interactions between FL(2)d, Ime4, and Mettl14 (28) as well as between Fl(2)d, SXL, and Vir (30, 31). The absence of SXL induces female-specific lethality, which is due, in part, to its failure to prevent dosage compensation in females (32). While the single amino acid substitution alleles fl(2)d1 and vir2F also lead to female-specific lethality, null mutants are lethal in both sexes, suggesting an additional function of Fl(2)d and Vir apart from the SXL-dependent gene regulation (27, 33). In contrast, Ime4-null mutants, lacking m6A marks in mRNA, remain viable and fertile (29), indicating that Fl(2)d and Vir have distinct functions, independent of the m6A pathway. Since our previous proteomic data revealed an evolutionarily conserved formation of the WTAP complex and its interaction with general splicing factors (15), here, we investigate whether the WTAP complex regulates AS in mammalian cells, independent or dependent on the m6A modifications.

In this study, we performed comprehensive analysis of AS events regulated by WTAP complex *via* RNAi coupled with RNA-seq analysis using HUVECs, in which we identified interacting proteins of WTAP using shotgun proteomics (15). We demonstrate that the WTAP complex major components WTAP, VIRMA, ZC3H13, and CBLL1 function as splicing regulators to promote exon skipping and intron retention, several of which occur in the last exon with competition between splicing and polyadenylation. Proteomic analysis of CBLL1-interacting proteins identified 3′-end processing factors: cleavage and polyadenylation specific factors CPSF1 and CPSF2, FIP1L1 and symplekin, suggesting a role for WTAP/CBLL1 in facilitating polyadenylation and determining the last exon. Further analysis of the sequences in the regulated introns/exons, as well as minigene analysis, revealed the involvement of G-quadruplex formation near 5′ and 3′ splice sites in AS regulation by WTAP, suggesting possible participation of the G-quadruplex-interacting/WTAP-interacting proteins, such as FMR1, FXR1, and FXR2.

## Results

### Knockdown of WTAP complex leads to differential gene expression and differential isoform expression related to cell cycle, morphogenesis, cell migration, and cell adhesion

**Figure 1:**
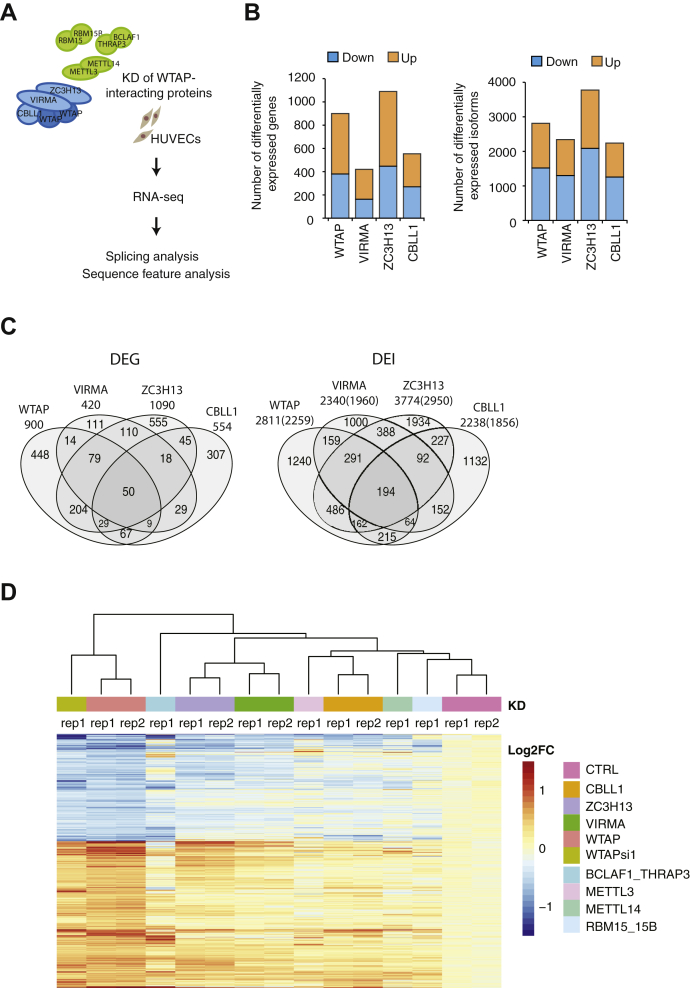
**The effect of knockdown of the WTAP complex major components on gene expression.***A*, schematic description of RNA-seq analysis to identify WTAP complex-regulated AS events. *B*, numbers of differentially expressed genes (DEGs) and differentially expressed isoforms (DEIs). Fold change ≥1.5 *versus* control siRNA-treated samples, FDR ≤0.1. *C*, overlap and numbers of DEGs and DEIs among KD of each of WTAP complex major components. *Numbers in parenthesis* of DEIs indicate corresponding numbers of genes. Statistical significance of overlap between two groups was assessed using Fisher’s exact test. All the combinations showed *p* < 0.0001. More information is provided in Fig. S3. *D*, heatmap and hierarchical clustering of differential expressed genes by WTAP-KD (n = 900 genes) showing a similar trend of gene expression. Values are indicated as Log2 fold change *versus* the average of control siRNA-treated samples. CTRL, control siRNA.

To determine the implications of the WTAP complex in gene expression and splicing regulation, we selected knockdown (KD) of WTAP complex major components (15) (WTAP, VIRMA, ZC3H13, and CBLL1, hereinafter referred to as the “major components”) in duplicates to obtain the overlapped events with higher confidence. RNA-seq on HUVECs treated with siRNA against the major components yielded on average ∼39 million uniquely mapped 150 nt pair-end sequencing reads of each library (Fig. 1*A*). KD efficiency was evaluated by immunoblot analysis (Fig. S1). Transcriptomic analysis with R software DESeq2 revealed 900, 420, 1090, 554 differentially expressed genes (DEGs) by KD of WTAP, VIRMA, ZC3H13, and CBLL1, respectively (FDR ≤0.01 and fold change ≥1.5, Fig. 1, *B* and *C*, left panel. TPM (transcripts per million) values are in Table S1). Gene ontology (GO) analysis of the DEGs showed enrichment of cellular processes linked to cell cycle, immune response, morphogenesis, and cell migration by WTAP, cell cycle and chemokine secretion by VIRMA, cell migration and the metabolic process by ZC3H13, and cell cycle, cell adhesion, and cell migration by CBLL1 (Fig. S2). Heatmap of the WTAP-regulated genes showed that majority of genes are similarly regulated by KD of VIRMA (74.7%), ZC3H13 (86.2%), and CBLL1 (73.0%) as well as KD of other WTAP-interacting proteins BCLAF1/THRAP3 (70.8%), RBM15/RBM15B (54.9%), METTL3 (57.4%), and METTL14 (59.0%) (one replicate each, as reference) (Fig. 1*D* and Fig. S3*A*). (Note, a similar trend was inferred when the change in gene expression was in the same direction as that of WTAP-KD and |Log2FC| ≥ 0.1).

Differentially expressed isoforms (DEIs) were evaluated with EBSeq software using transcript estimated counts obtained with the RSEM software (https://deweylab.github.io/RSEM/), which is capable of detecting DEIs regulated by alternative splicing and/or alternative polyadenylation, as well as DEIs regulated by transcription and/or RNA stability. As expected, DEI analysis identified larger number of genes as differentially expressed, showing 1518, 1298, 2086, and 1255 isoforms (corresponding to 1261, 1129, 1660, and 1061 genes) that were upregulated by KD of WTAP, VIRMA, ZC3H13, and CBLL1, respectively, whereas the expressions of 1293, 1042, 1688, and 983 isoforms (corresponding to 1103, 952, 1459, and 880 genes) were downregulated by KD (FDR ≤0.01 and fold change ≥1.5, Fig. 1, *B* and *C*, right panel and TPM values are in Table S2). Regulation of the *WTAP* transcript by the WTAP complex (VIRMA, ZC3H13, and CBLL1) *via* competition between splicing and polyadenylation was also observed by DEI analysis, consistent with our previous study (Fig. S3*B*). Approximately 72% of the DEGs were also detected by DEI analysis. GO analysis of the DEIs showed similar enrichment of GO terms compared with the DEGs (Fig. S2).

### Exon inclusion is the most frequent splicing alteration after KD of WTAP complex major components

**Figure 2:**
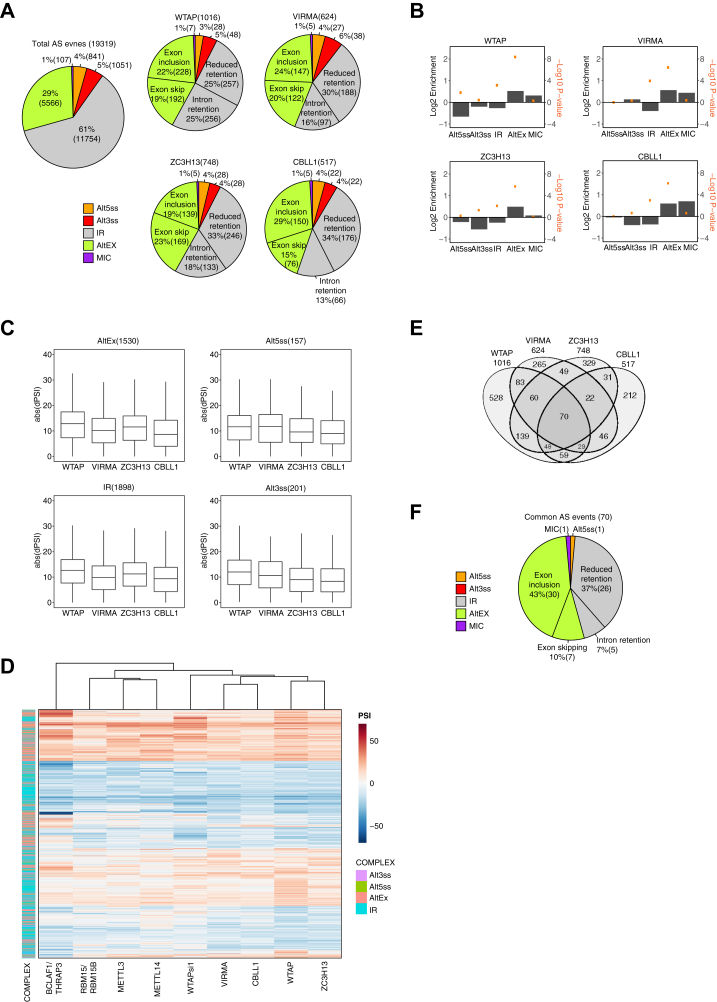
**RNA-seq analysis of alternative splicing alternations by KD of WTAP complex.***A*, distribution of categories of alternative splicing (AS) events differing in each KD sample *versus* control siRNA-treated sample. The overall distribution of the AS event categories showed 10 < PSI (Percent spliced-in) < 90 in at least one of the compared samples. Numbers of each class of event are indicated in *parenthesis*. *B*, Log_2_ values of fold enrichment (relative to the background frequency of annotated AS events) and −log_10_ of *p*-values (Fisher’s exact test) are provided for each AS category. *C*, the distribution range of ΔPSI by each KD (3792 AS events with |ΔPSI | ≥ 10 in at least one KD were extracted). The number of AS events is indicated in *parenthesis*. *D*, heatmap and hierarchical clustering of affected AS events with ΔPSI ≥15 by KD of at least one of the major components (n = 1970 events). *E*, overlap and number of AS events among KD of the WTAP complex major components. *F*, distribution of categories of overlapping alternative splicing (AS) events among WTAP, VIRMA, ZC3H13, and CBLL1 knockdowns. Alt3SS, alternative 3′ splice sites; Alt5SS, alternative 5′ splice sites; AltEx, alternative exons; IR, intron retention; MIC, microexons.

We next examined AS using the vast-tools software, which detects splicing events of five categories based on the database: alternative 5′ splice site (Alt 5′ss), alternative 3′ splice site (Alt 3′ss), alternative (cassette) exon skipping/inclusion (Alt Ex), microexons (MIC), and intron retention (IR). In total, 1016, 624, 748, and 517 AS events were identified as affected with a minimum difference in percent spliced-in (ΔPSI) of 15% by KD of WTAP, VIRMA, ZC3H13, and CBLL1, compared with control siRNA-treated samples, respectively (Fig. 2*A* and Table S3). All categories of AS events were affected (Fig. 2*A*); most of the events corresponded to cassette exon skipping/inclusion and IR in all KD samples. However, once normalized according to the overall distribution of AS events (identified as AS when at least one of the compared samples shows 10 < PSI < 90, Fig. 2*A* left), cassette exon skipping/inclusion was most enriched (Fig. 2*B*). KD of WTAP and ZC3H13 showed higher ΔPSI than that of VIRMA and CBLL1 in AltEx and IR (Fig. 2*C*). Comparing AS events among the major components KD samples, AS events were generally found to change in same direction in most cases (∼73%, 5% > ΔPSI in the same direction) as shown in the heatmap of ΔPSI of AS events in which at least one KD sample showed >15% of ΔPSI (1970 events, Fig. 2*D* and Fig. S4*A*). Similar trends were also observed by KD of other WTAP-interacting proteins, BCALF1/THRAP3, RBM15/RBM15B, METTL3, and METTL14 (∼65%, one replicate each, as reference). Seventy events were commonly affected by KD of all four major components (Fig. 2*E*) with ΔPSI ≥15%. Interestingly, reduced IR (37%) and cassette exon inclusion (43%) are the most affected AS categories by KD, indicating that WTAP complex inhibits splicing (Fig. 2*F*). There were 229 commonly regulated events with ΔPSI ≥15% in at least three of the major components exhibiting the similar distribution of the AS categories (Fig. S4*B*).

**Figure 3:**
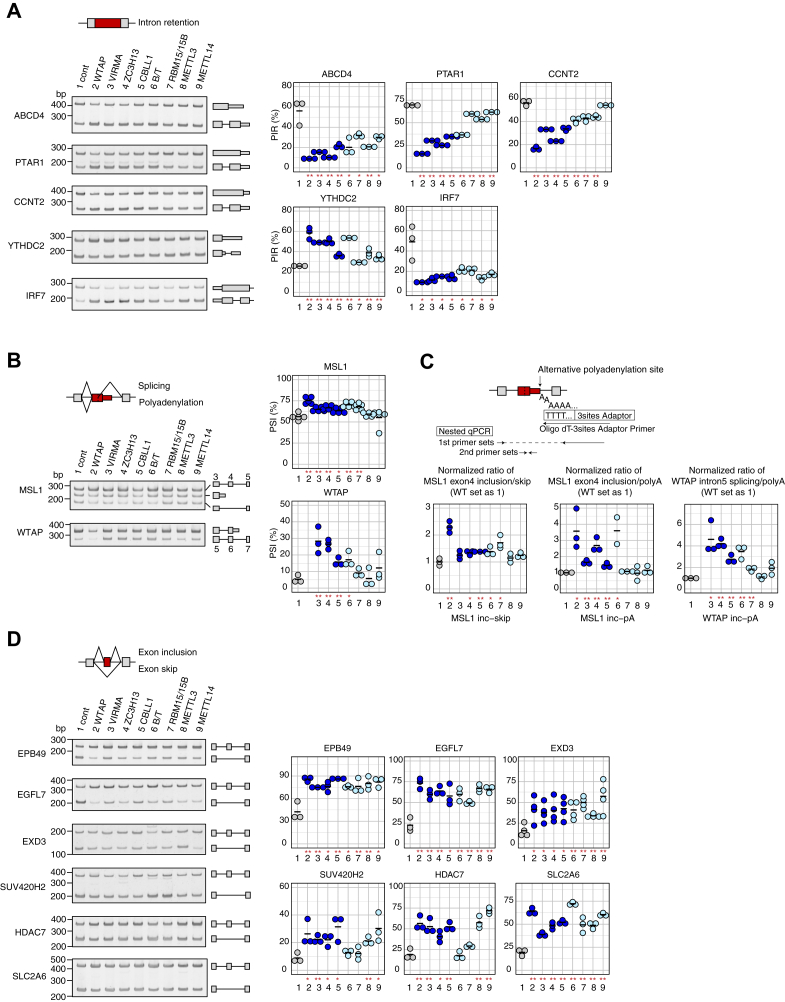
**WTAP complex major components promote intron retention, polyadenylation, and exon skipping.** Predicted AS changes by KD of WTAP complex were validated using RT-PCR: (*A*) intron retention, (*B*) polyadenylation and splicing, and (*D*) exon skipping. Predicted AS changes by KD of WTAP complex were validated using RT-PCR. *Left*, representative gel image of RT-PCR. The PCR products were stained with SYBR Gold Nucleic Acid Gel Stain (Thermo Fisher). The drawing *right side* of the image indicates the corresponding spliced/unspliced products. *Rectangle*, *thin rectangle*, and *line* correspond to an exon, 3′ UTR, and introns, respectively. *Right*, dot plot of the percent of exon inclusion (Percent spliced-in; PSI) or intron retention (Percent intron retention; PIR) calculated using the molarity data from the Bioanalyzer. The *horizontal lines* represent the average from three independent experiments performed in HUVEC, except for MSL1, five independent experiments, and EXD3, four independent experiments. The x-axis represents the following: 1. Control siRNA, 2. WTAP KD, 3. VIRMA KD, 4. ZC3H13 KD, 5. CBLL1 KD, 6. BCLAF1/THRAP3 KD, 7. RBM15/RBM15B KD, 8. METTL3 KD, and 9. METTL14 KD. ∗∗*p* < 0.01, ∗*p* < 0.05 *versus* control siRNA-treated samples (*t* test). *C*, ratios of exon inclusion to skip and inclusion to alternative polyadenylated products in WTAP complex KD samples relative to the values in control samples, as determined using nested-qPCR from three independent biological replicates. The *horizontal lines* represent the average. cont, control siRNA; inc, inclusion; pA, alternative polyadenylation.

As shown in Figure 3, the RT-PCR assays validated several AS events predicted as commonly regulated by the four major components. Several intron retention events (ABCD4, PTAR1, CCNT2, and IRF7 genes) were reduced by KD of the major components as well as the other WTAP-interacting proteins, BCLAF1/THRAP3, RBM15/15B, METTL3, and METTL14, while the opposite was also observed in the case of the *YTHDC2* gene (Fig. 3*A*). The regulated intron retentions were often observed in the last exon (Fig. 3*A*, described below). Given that most of the affected IR is reduced by KD of WTAP complex, IR observed in YTHDC2 gene might be regulated indirectly, such as *via* other protein regulated by WTAP complex. In the case of *MSL1* and *WTAP* genes, splicing was increased instead of alternative polyadenylation, leading to the formation of longer isoforms by KD of WTAP complex major components (Fig. 3*B*). We performed 3′ rapid amplification of cDNA ends (RACE) to isolate the alternative polyadenylation isoforms of *MSL1* and *WTAP*. Sequencing the 3′ end of the mRNAs revealed an alternative polyadenylation event involving a poly(A) signal located in intron 3 and intron 5, respectively. Significant increase in ratio of exon inclusion and polyadenylated products were detected in *MSL1* and *WTAP* transcripts by KD of the major components, suggesting that WTAP complex might promote polyadenylation (Fig. 3*C*). Increased exon inclusion was also validated in *EPB49*, *EGFL7*, *EXD3*, *SUV420H2*, *HDAC7*, and *SLC2A6* genes (Fig. 3*D*).

### WTAP complex represses splicing of short introns with a high GC content

**Figure 4:**
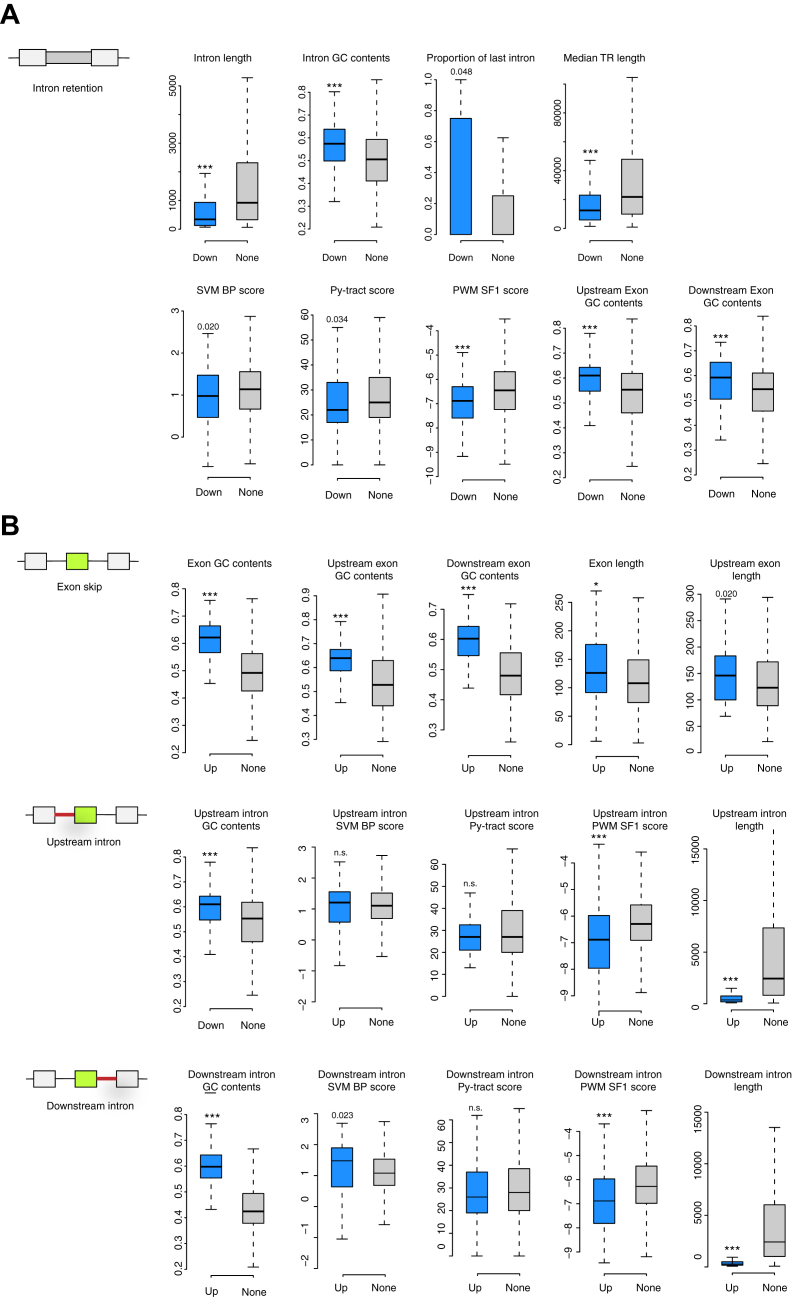
**Analysis of transcript features associated with WTAP complex-regulated AS events.***A*, sequence features of reduced retained introns by KD of WTAP complex and nondifferentially spliced introns compared with the control samples. *Boxplots* represent the distribution of feature values in the reduced retained introns (Down) (ΔPIR ≤ −15) and nondifferentially spliced introns (None) (|ΔPIR| ≤ 5). *B*, sequence features of alternative exons increased by KD of WTAP complex and nondifferentially spliced alternative exons compared with the control samples. *Boxplots* represent the distribution of feature values in the increased (Up) (ΔPSI ≥15) and nondifferentially spliced alternative exons (None) (|ΔPSI| ≤ 5). Outliers were discarded, *boxes* indicate interquartile range (IQR), whiskers extend to 1.5 times IQR. *Black lines* indicate median values. ∗*p* < 0.01, ∗∗*p* < 0.001, ∗∗∗*p* < 0.0001, Mann–Whitney *U*-test (two-sided).

To examine sequence elements associated with the regulated AS events by the WTAP complex, common AS events that were affected by KD of at least three of the major components were analyzed using Matt software (http://matt.crg.eu/). Analysis of sequence feature of decreased retained introns (91 introns) compared with nonaffected retained introns (*i.e.*, 3247 introns of 10 < percent intron retention (PIR) of control sample < 90, but not affected by WTAP complex KD) revealed that the introns more spliced by KD of WTAP complex are significantly short, more GC contents, and enriched in the last intron (Fig. 4*A*). The 3′ss displayed weaker branch points, as determined by SVM-BPfinder and SF1 binding site scores (34, 35, 36, 37) and weaker polypyrimidine tracts (Fig. 4*A*). Flanking upstream and downstream exons of the retained introns were also GC-rich (Fig. 4*A*).

Analysis of sequence feature of the increased exon inclusion (75 exons) compared with the nonaffected exon skipping/inclusion events (*i.e.*, 978 exons of 10 < PSI of control sample < 90, but not affected by WTAP complex KD) revealed that the exons that were more included by KD of WTAP complex were more GC-rich and marginally longer compared with nonaffected exons (Fig. 4*B*). The flanking upstream and downstream introns display significantly higher GC contents, weaker branch points, and are significantly shorter compared with those of nonaffected exons (Fig. 4*B*).

Taken together, these results indicate that the WTAP complex represses splicing of the introns with a short length and higher GC contents. The relation of GC content and intron length has been described. Bioinformatic study on gene structure across various eukaryotes revealed that the mammalian genomes exhibit two groups of exons: GC-rich exons flanked by short introns and GC-poor exons flanked by long introns with lower GC content than the exons, which may be recognized by the splicing machinery under the different mechanism: intron and exon definition (36). It, therefore, appears that the WTAP complex is involved in the regulation of the former group of the exons.

### WTAP regulates SLC2A6 exon inclusion through potential G-quadruplex forming sequences

**Figure 5:**
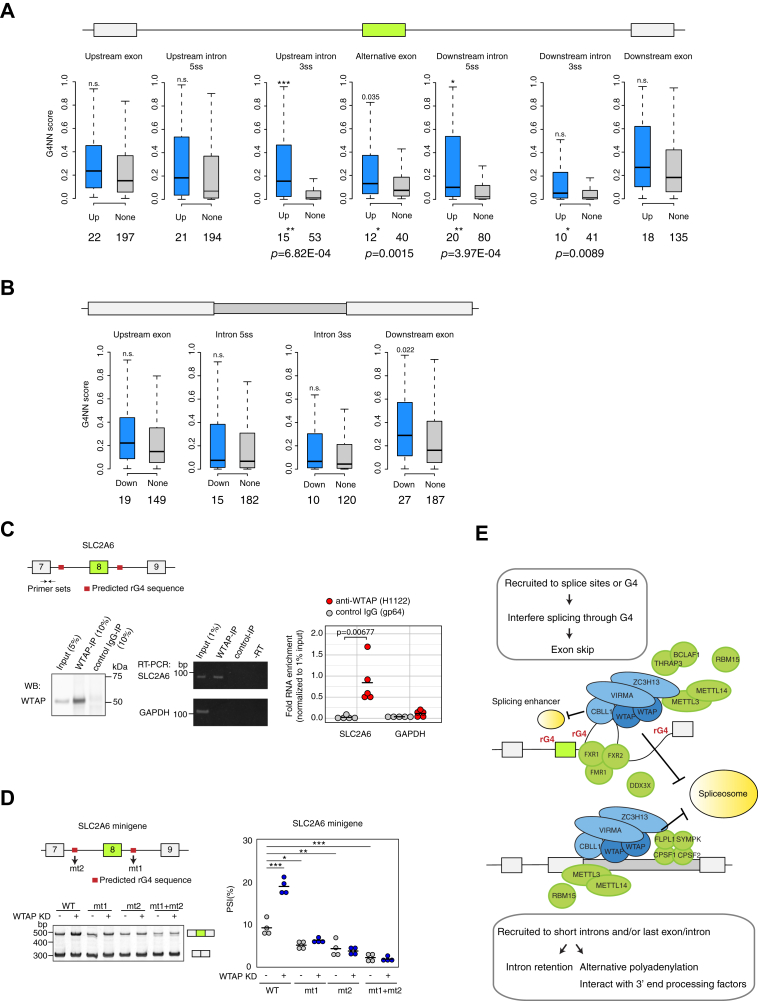
**WTAP regulates alternative splicing of SLC2A6 *via* rG4 sequences.***A*, G4NN scores of the included exons (n = 75) by KD of WTAP complex and nondifferentially spliced exons (n = 978). Upstream and downstream exon sequences and 100 nucleotides of 5′ss or 3′ss of flanking introns of the regulated exons were used for the analysis. *Boxes* indicate interquartile range (IQR), whiskers extend to 1.5 times IQR. Outliers, which lie beyond the extremes of the whiskers, were excluded. Significant differences were evaluated using Welch’s ANOVA followed by Games–Howell post hoc pairwise comparison (∗*p* < 0.01, ∗∗*p* < 0.001, ∗∗∗*p* < 0.0001). *Bottom*, the number of exons/introns with >0.5 G4NN score and *p*-values (Fisher’s exact test) are indicated. *B*, G4NN scores linked to the decreased retained introns (n = 90) by KD of WTAP complex and nonaffected introns (n = 1000) are indicated; 100 nucleotides of 5′ss or 3′ss of intron, upstream exon, and downstream exon. *C*, the interaction of WTAP and SLC2A6 RNA was determined using RNA Coimmunoprecipitation (RIP) and quantitative RT-PCR. HUVECs were cross-linked with 1% formaldehyde, and the protein/RNA complexes cross-linked to WTAP were immunoprecipitated with anti-WTAP (H1122) antibody or anti-viral gp64 protein antibody (as control). RNA was extracted from the immunoprecipitants (IPs) following reverse cross-linking and used for RT-PCR analysis (*left*). Western blot analysis of WTAP protein in IPs. 5% input and 10% IP were loaded. *Middle*, representative gel image of RT-PCR from the RIP samples using SLC2A6-or GAPDH-specific primers. *Right*, the interaction of WTAP with SCL6A6 transcripts was determined using RIP-qPCR. GAPDH was used as a negative control. *Horizontal black lines* represent the average of five independent experiments; *p*-value *versus* control IgG (*t* test). *D*, minigene splicing assays showing that rG4 sequences influence exon inclusion. *Left*, diagram of the exon/intron structure of the genomic region included in the *SLC2A6* minigene and RT-PCR analysis of minigene-derived transcripts isolated from HUVEC transfected with the indicated constructs (WT, WT sequence; mt1, a mutation in G4 of intron 8; mt2, a mutation in G4 of intron 7; mt1+mt2, double mutations of G4 both in downstream and upstream introns). The positions of different spliced products are indicated by drawing, on the *right side* of the gel image. *Right*, quantification of the percentage of exon 8 inclusion for five independent biological replicates. Significant differences were evaluated using one-way ANOVA/Tukey HSD post hoc test (∗*p* < 0.01 ∗∗*p* < 0.001, ∗∗∗*p* < 0.0001). *E*, model: Inhibitory effect of the WTAP complex on splice site recognition. Exon skipping, the WTAP complex is recruited to splice sites *via* the interaction between rG4-binding proteins and rG4 sequences located in the flanking introns of the alternative exon (or recruited to other region *via* other RNA binding protein of WTAP complex) and interferes with the splice sites recognition through rG4 sequences, thereby leading to exon skipping. In the case of SLC2A6, rG4s promote exon inclusion possibly *via* the structure or other RBPs of the splicing enhancers, which WTAP inhibits or competes with. Another possibility is that the WTAP complex may sequester rG4 binding proteins, which are involved in splice site recognition. Exon skipping by the WTAP complex results in generating truncated/nonfunctional isoform, thus regulates the expression of the full-length protein. Intron retention, the WTAP complex is recruited to the last exon and inhibits splicing inside the last exon, as well as promotes polyadenylation. WTAP/CBLL1 would promote polyadenylation by recruiting the 3′end-processing factors such as CPSFs. Splicing in the last exon changes the C-terminus structure of the protein or affects mRNA stability and thus protein expression.

The observed enrichment of GC-rich sequences in WTAP-regulated exons/introns prompted us to further examine the involvement of G-quadruplexes in AS regulation, as WTAP interacts with G-quadruplex interacting proteins, such as FMR1, FXR1, and FXR2 (15). G-quadruplex (G4) is formed within guanine-rich sequences, where four G-tracts of two or more guanines, separated by short stretches of other nucleotides, are assembled in a square planar arrangement *via* Hoogsteen hydrogen bonding. RNA G-quadruplexes (rG4) are involved in regulating AS as *cis*-elements to recruit splicing factors (37, 38, 39). Analysis of the potential G4 forming sequence using the G4RNA screener (40) revealed that the 3′ss and 5′ss of the flanking introns of the regulated exons display significantly higher G4 scores (Fig. 5*A*). Introns with ≥0.5 G4 score, the default threshold proposed for the G4 RNA screener, were observed more frequently (The number of the introns/exons and *p*-values assessed using Fisher’s exact test are shown in Fig. 5*A*). In contrast, no difference in G4 score and the number of introns/exons with ≥0.5 G4 score were observed in the 5′ss and 3′ss of the decreased retained introns (Fig. 5*B*). All G4 sequences in WTAP-regulated exons and introns are listed in Table S4 with the threshold ≥0.5.

To determine the relevance of rG4 in WTAP-regulated splicing, we examined the binding of WTAP complex to the target RNA containing the potential G4 sequence located in the flanking region of the regulated exon (Fig. 5*C*). SLC2A6, in which we validated the regulated exon inclusion by KD of WTAP complex (Fig. 3*D*), contains a potential G4 sequence at 5′ss of upstream intron and 5′ss of the downstream intron with G4 score 0.92 and 0.95, respectively (Fig. 5*C* and Table S4). RNA Coimmunoprecipitation (RIP) and quantitative RT-PCR demonstrated the direct binding of WTAP complex to SLC2A6 transcript (GAPDH was used as negative control), suggesting that the WTAP complex regulates SLC2A6 alternative splicing directly.

Further analysis with SLC2A6 minigene was performed using genomic regions of *SLC2A6* spanning exons 7 to exon 9. RT-PCR analyses using minigene-specific primer pairs confirmed that splicing from the SLC2A6 minigene was WTAP-dependent, as KD of WTAP resulted in an increase of exon inclusion (from 9.4 to 19.2%) (Fig. 5*D*, lanes 1 and 2). We performed a mutational analysis of the predicted G4 by substituting guanine nucleotides with other nucleotides (See Supporting information) to abolish G4 formation. The G4 mutant of the downstream intron (referred to as mt1) and the upstream intron (referred as mt2) resulted in the decrease of exon inclusion (from 9.4 to 5.2 and 4.4%, respectively), indicating these G4s enhance exon inclusion (Fig. 5*D* lanes 1, 3, and 5). The WTAP-dependent effect was abolished in mt1 and mt2 (Fig. 5*D* lanes 3 and 4, and 5 and 6), indicating that WTAP-dependent AS involves these G4s. The G4 double mutant (referred to as mt1+mt2) showed more decrease in exon inclusion (from 9.4 to 2.3%) than that of a single mutant and no WTAP-dependent effect. Taken together, our results indicate that both G4s in the upstream and the downstream introns of *SLC2A6* minigene promote exon inclusion possibly *via* the structure or other RBPs of the splicing enhancer, which WTAP inhibits or competes with, thus WTAP-KD leads to an increase of exon inclusion (model Fig. 5*E*).

### CBLL1 and WTAP shares most interacting proteins including a 3′-end processing complex

**Figure 6:**
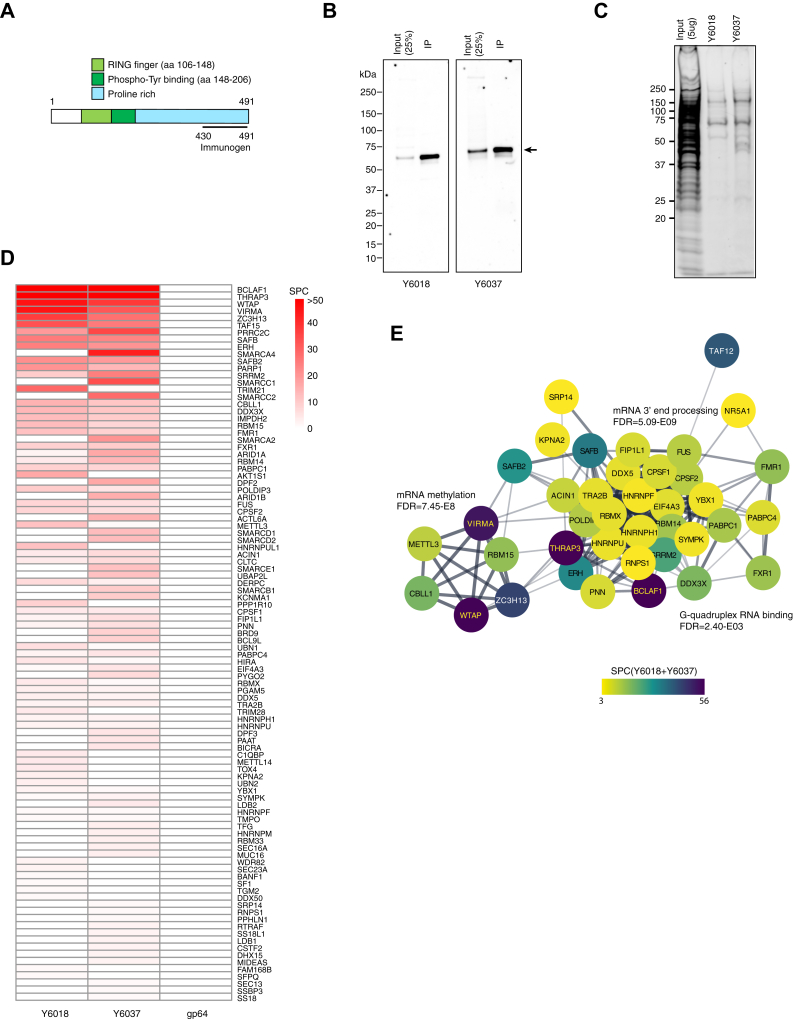
**Immunoprecipitation of CBLL1 complexes using high-affinity monoclonal antibodies.***A*, domains and unique amino acid repeat in the CBLL1 protein. It contains a RING finger and phosphor-tyrosine binding motif at the N-terminus, and unique proline-rich repeats that span most of the C-terminal region. Specific monoclonal antibodies were generated using the antigen in the C-terminal region indicated with a *bold line*. *B*, the specificity of anti-CBLL1 antibodies. Whole lysates of HUVECs were subjected to immunoblot analysis using Y6018 and Y6037 antibodies. The *arrow* indicates the band corresponding to CBLL1, which is efficiently immunoprecipitated by each antibody. *C*, the SYPRO Ruby stain of the immunopurified CBLL1 and its interacting proteins from HUVECs. *D*, the proteomic profile of CBLL1-interacting proteins. The values represent the total spectrum counts (SPC). *E*, network of physical relationships (edges) of the top 56 identified proteins isolated with at least one unique peptide by the anti-CBLL1 antibodies, Y6016 and Y6037, but not with the negative control antiviral protein antibody (gp64). The illustration and enrichment analysis were performed using Cytoscape version 3.8.0.

Our finding that WTAP-regulated IR events are enriched in the last exon suggests the interplay of WTAP complex with 3′ end processing complex, consistent with the previous results that a majority of m6A residues are present in the last exons, exerting a role in 3′ UTR regulation including poly (A) choice (8). Indeed, the WTAP-interacting-protein VIRMA has recently been shown to associate with polyadenylation cleavage factors CPSF5 and CPSF6 and affects alternative polyadenylation (22). The 3′ end processing events include cleavage at a specific site in the pre-mRNA followed by the addition of the poly(A) tail, which stimulates translation from the mRNA and transport of the mRNA to the cytoplasm and protects the mRNA from degradation. Interaction between splicing factors and 3′ end processing factors, as well as transcription machinery, is supposed to be required for exon definition for the last exon (41, 42). To further dissect the protein–protein network of the WTAP complex, we expanded our proteomic analysis to the other major component, CBLL1. Anti-CBLL1 monoclonal antibodies (Y6018 and Y6037) were generated against the C-terminal sequence of the human CBLL1 protein corresponding to aa 430 to 491 (Fig. 6*A* and Experimental procedures). The specificity for CBLL1 was tested by immunoblotting. Endogenous CBLL1 corresponding to a ∼55 kDa band was detectable and immunoprecipitated effectively by each antibody (Fig. 6*B*). From the results of the protein staining of the immunoprecipitates, the purification quality was regarded as sufficient to carry out shotgun proteomics (Fig. 6*C*). Using these antibodies, we performed shotgun proteomics of the endogenous CBLL1-interacting proteins from whole cell lysates of HUVECs (Fig. 6*D*). In addition to the WTAP complex major components WTAP, VIRMA, and ZC3H13, and transient components BCLAF1, THRAP3, RBM15, and METTL3, the identified proteins included 3′-end processing factors; cleavage and polyadenylation-specific factors CPSF1 and CPSF2, FIP1 and symplekin, as well as rG4 interactors; FMR1, FXR1, FXR2, and DDX3X, TET family of RNA-binding protein TAF15, and enhancer of rudimentary homolog (ERH) and its interacting proteins, polymerase delta-interacting protein 3 (POLDIP3) and scaffold attachment factor B1 (SAFB1) (Fig. 6*D* and Table S5). Interestingly, most of the identified proteins (75%) overlap with those of WTAP (15) (Fig. S5*A*). Supporting this, immunoprecipitation and immunoblotting analyses indicated that nearly all WTAP proteins immunoprecipitate with CBLL1, and vice versa (Fig. S5*B*), indicating the stable interaction of these two proteins. Furthermore, co-IP experiments using V5- or FLAG-tagged WTAP reveal a multimer formation of WTAP (Fig. S5*C*). This result is consistent with the higher yield of total spectrum counts for WTAP protein in CBLL1-immunpprecipitates (Fig. 6*D*). As shown in Figure 6*E* of the protein–protein network for CBLL1-interacting proteins, GO terms, including “RNA splicing” (FDR = 3.41E-24), ”mRNA methylation” (FDR = 7.45E-8), and ”3′ end processing” (FDR = 5.09E-9), are enriched. Taken together, our findings demonstrate that CBLL1 and WTAP stably form a complex that interacts with splicing factors, the rG4-interacting proteins, and 3′ end processing factors, suggesting a possible role in recruitment of the polyadenylation complex, as well as splicing regulation, which might promote polyadenylation and define the last exon, competing with splicing. KD of WTAP may fail to recruit polyadenylation factors and define the last exon, thus splice sites inside the last exon can be activated and spliced (Fig. 3, *A* and *B*).

### Alternative splicing of SUV420H2 affects cell cycle progression

#### Delay of H4K20me1 accumulation and G2/M transition in WTAP-KD HeLa cells and SUV420H2 overexpressed cells

**Figure 7:**
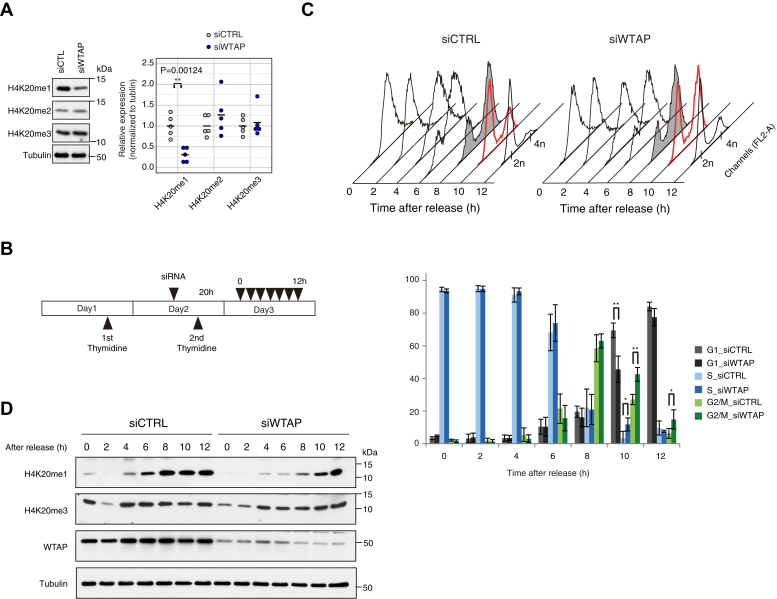
**The effect of *WTAP* knockdown on histone H4K20 methylation state during the cell cycle.***A*, relative levels of H4K20me1/me2/me3 with or without depletion of WTAP were detected by immunoblotting with H4K20 methylation state-specific antibody. The results were quantified and are shown in the *right panel*. The values are the average of three independent experiments; ∗∗*p* < 0.01 (*t* test). *B*, experimental design. HeLa cells were synchronized with a double thymidine block, and the siRNAs were transfected at the indicated time. Cells were analyzed every 2 h after the release from the thymidine block. *C*, FACS analysis of the cells at each time point as indicated at the *top*. The data are represented in a histogram (*Top*) and the distribution (%) of each phase is shown (*Bottom*). The values are the average of four independent experiments; ∗∗*p* < 0.01, ∗*p* < 0.05 *versus* control (*t* test). Error bars, SD. *D*, the Western blot analysis of H4K20me1 and H4K20me3 in synchronized HeLa cells using the antibodies indicated on the *left*. Alpha-tubulin was used as a loading control.

Furthermore, we examined the biological significance of AS events regulated by the WTAP complex. Exon3 skipping of *SUV420H2* gene, which harbors continuous runs of guanine in the upstream of the alternative exon, introduces premature stop codon and would lead to transcript degradation due to nonsense-mediated decay. As shown in Figure 3*D*, KD of the WTAP complex increases the protein coding transcript of *SUV420H2*. Since SUV420H2 is a histone-lysine methyltransferase, which generates the di- and trimethyl lysine 20 of histone H4, *i.e.*, H4K20me2 and H4K20me3, we examined the effect of WTAP depletion on histone H4 methylation. As a result, the immunoblot analysis revealed that the global level of H4K20me1 was decreased in the WTAP-KD cells compared with the control cells, whereas no significant difference in the levels of H4K20me2 and H4K20me3 was observed between the control and WTAP-KD cells (Fig. 7*A*), A previous study on Histone H4 modification dynamics during cell cycle has reported that H4K20me2 is much more abundant, >3- to 15-fold relative to H4K20me1 (43), which can explain why the increase in the level of H4K20me2 from H4K20me1 was not detected while the decrease in the level of H4K20me was.

#### H4K20 methylation is regulated during the cell cycle

H4K20me1 oscillates during the cell cycle, and maintenance of the H4K20me levels is critical for proper cell cycle progression through its protective roles in genome stability, DNA replication, mitotic condensation, and transcription (reviewed in Beck *et al.* (44)). We set out to investigate the effect of H4K20me1 reduction by WTAP-KD during the cell cycle using HeLa cells synchronized with a thymidine double block (Fig. 7*B*), as we confirmed that the AS regulation of SUV420H was also observed in the HeLa cells (Fig. S6*A*). FACS analysis revealed that, upon KD of WTAP, HeLa cells showed a delay at the G2/M phase after release from the thymidine block compared with the control cells between 8 and 12 h (Fig. 7*C*), which can be detected by the G2/M accumulation observed in WTAP-KD cells at 10 and 12 h and the subsequent delay of the G1 phase transition at 10 h. In addition, the delay of accumulation of H4K20me1 was observed upon WTAP-KD (Fig. 7*D*), although control cells showed the accumulation of H4K20me1 from late S (6 h after release) into G2/M (8 h) at the highest level. The change of the level of H4K20me3 was not detected by WTAP-KD during the cell cycle.

**Figure 8:**
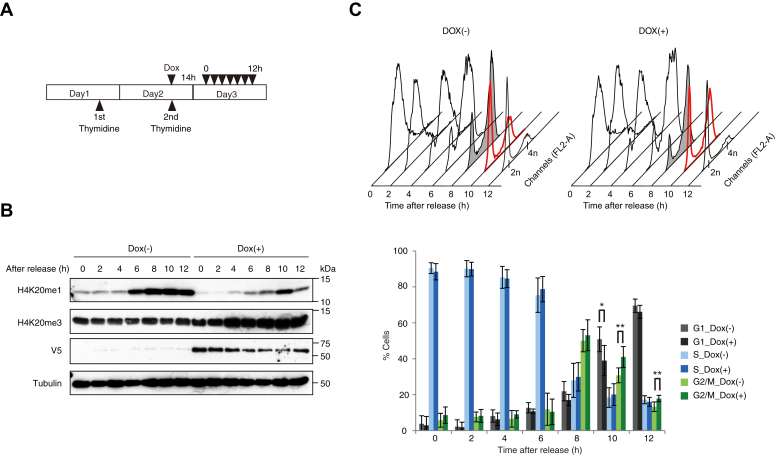
**The effect of SUV420H2 overexpression during the cell cycle.***A*, experimental design. Tetracycline-inducible HEK293 stable cell *lines* expressing a V5-tagged SUV420H2 were subjected to cell cycle synchronization analysis in the absence or presence of doxycycline at the concentration of 100 ng/ml, 14 h before release. *B*, FACS analysis of the cells every 2 h after the release. The data are represented with a histogram (*Top*) and the distribution (%) of each phase is shown (*Bottom*). The values are the average of seven independent experiments; ∗∗*p* < 0.01, ∗*p* < 0.05 *versus* control (*t* test). Error bars, SD. *C*, the Western blot analysis of H4K20me1 and H4K20me3 in synchronized HEK293 cells using the antibodies indicated on the *left*. Alpha-Tubulin was used as a loading control.

Then we tested whether the substantial expression of SUV420H2 caused the delay in the G2/M phase by using tetracycline-inducible HEK293 stable cell lines expressing a V5-tagged SUV420H2 synchronized with thymidine double block (Fig. 8*A* and Fig. S6*B*). As expected, the decrease in H4K20me1 level and the increase in H4K20me3 level were observed in dox-treated cells compared with the control cells (Fig. 8*B*). FACS analysis revealed that dox-induced SUV420H2 overexpressed cells also exhibited a delay in the G2/M phase and subsequent G1 transition at 10 h after release from the thymidine block compared with dox (−) cells (Fig. 8*C*). These findings suggest that delayed accumulation of H4K20me1 by WTAP-KD might contribute to the delay in the G2/M phase.

## Discussion

Our findings demonstrate that WTAP and its interacting proteins, VIRMA, ZC3H13, and CBLL1 have a distinct role in splicing regulation, *i.e.*, inhibitory effect in splicing of a subset of genes with short introns and high GC content, for several substrates, in a G-quadruplex sequence-dependent manner. The secondary structure of RNA greatly impacts posttranscriptional regulation *via* interaction with proteins (45). As such, high-throughput sequencing techniques have recently been applied for transcriptome-wide identification of RNA structure (46), including the rG4 (47, 48, 49), which has greatly expanded our knowledge on the biological roles played by RNA secondary structure. Moreover, emerging evidence has indicated the important role of rG4 formation in regulating RNA metabolism, including translational regulation (50), 3′ end processing (51), and AS (39, 52), and has, therefore, been reported as associated with neurodegenerative disease (53) and cancer (54). Several RNA-affinity approaches for identifying rG4-binding proteins have revealed that RGG and RGG-like motifs are enriched for binding (52, 55). Among them, VIRMA, FXR proteins (FMRP, FXR1P, and FXR2P), and DEAD-box helicases DDX3X and DDX5, represent the most likely candidates to function as linkages between rG4 and AS regulation by the WTAP complex, as shown in our previous and current proteomic analysis of WTAP- and CBLL1-interacting protein (Fig. 6*D* and Fig. S5*A*). Although the molecular function of VIRMA is not well explored, the genetic and physical interaction with WTAP has been clearly demonstrated in *Drosophila* and mammalian cells, while a recent affinity-enrichment study identified human VIRMA as an rG4 interactor containing an RGG-like motif (52). DDX3X and DDX5 are also identified in the study, the latter of which has a reported G4-relevant role in splicing (56). Another candidate, FMRP, is known to primarily regulate translation efficiency under stress granules; however, recent studies have also reported nuclear localization of the FMRP isoform (57) and a role in AS (58, 59). Thus, it is possible that WTAP modulates rG4-interactors to the splice site with G4. Another possibility is that the WTAP complex may be recruited to splice sites *via* rG4. We examined the interaction of WTAP with the transcript from SLC2A6 minigene used in Figure 5*D*, to determine whether the mutation of the potential G4 sequence affects the binding. We observed the binding of WTAP to WT *SLC2A6* from the minigene and the double mutants (mt1+mt2) *SLC2A6* (data not shown), which indicated that the G-quadruplex is necessary for the AS regulation of *SLC2A6* by the WTAP complex but not for the interaction. WTAP might have been recruited to other region of SLC2A6 where it exerts an inhibitory effect *via* the G4 sequences located close to the binding sites, by recruiting or modulating G4 binding proteins.

Given that the amino acid sequence of WTAP contains a glutamine-rich region, leucine repeats, and coiled-coil structures in the middle section, corresponding to the section responsible for METTL3 and METTL14 binding (14) and self-interaction (Fig. S5*C*), as well as a disordered region in the C-terminus (analyzed using IDR, http://bioinf.cs.ucl.ac.uk/psipred/, Fig. S5*D*), while lacking RNA-binding domains, it is conceivable that WTAP functions as a hub protein for protein recruitment. The binding proteins may then serve as target determinants for the posttranscriptional regulation. The potential mechanisms by which the WTAP complex represses splicing are as follows: (1) binding to rG4 *via* VIRMA; (2) modulation of splicing factors through rG4, such as FXR proteins and DEAD-box helicases; (3) recruitment of polyadenylation complex; (4) m6A modification (*via* interaction with METTL3/METTL4); (5) a combination of these mechanisms. Interestingly a recent bioinformatic study revealed the colocalization of potential rG4 forming sequences and m6A at pre-mRNA splice sites, suggesting these features may function together in AS (60). VIRMA or other G4 interactors may bind to the target rG4, the recruited METTL3/METTL14 methylate nearby adenosine in the consensus sequence, and the resulting structure may subsequently impact the recognition of splice sites by the spliceosome. Determination of binding proteins for each target RNA, as well as the composition of the WTAP complex at the specific target, will serve to elucidate the detailed mechanism carried out by the complex.

Analysis of H4K20 methylation during the cell cycle showed that WTAP depletion resulted in the global decrease of H4K20me1 and delayed cell cycle progression. H4K20me1 is a histone marker that has been implicated in cell cycle regulation. H4K20me1 oscillates during the cell cycle and functions to protect genome stability, DNA replication, mitosis, and transcript; hence, maintenance of its abundance is critical for proper cell cycle progression (reviewed in (44)). H4K20me1 becomes modestly elevated during the S phase; however, it is markedly accumulated during early mitosis (43), which might be implicated in mitotic condensation by binding to condensin II (61). Knockout mouse studies have revealed that depletion of PR-Set7, which catalyzes H4K20me1, leads to accumulation within the G2/M of the cell cycle (62). Meanwhile, H4K20me3 exhibits only modest changes during the cell cycle (61, 63) and is involved in heterochromatin formation and gene silencing. The decrease in H4K20me1 by WTAP depletion is not likely caused by G2/M accumulation, as the level of H4K20me1 in G2/M rich cells would be expected to be greater than that in G1-rich control cells (See Fig. 7*C*). A recent study on the global coordination of AS and the cell cycle has identified 1300 genes with cell cycle-dependent AS changes (64). Ten genes (HDAC7, ELMOD3, MAN2V1, IMMP1L, IFT122, PKD1, ARIH2, LIAS, SLC17A9, and TUBGCP6) were also detected in our AS events identified as common events by the KD of the major components. Since WTAP protein expression varies with the cell cycle (16), even though WTAP expression was maintained at a certain basal expression level throughout the cell cycle, it is possible that WTAP is involved in the periodic AS regulation of these genes. Taken together, in addition to the destabilization of cyclin A2 mRNA, the change in histone H4K20 methylation states could cause the defect and/or delay in the cell cycle progression observed in WTAP-depleted cells.

Interestingly, our analyses also revealed that WTAP regulates AS of MSL1, a component of histone H4 at lysine-16 acetyltransferase. In *Drosophila*, SXL represses the expression of the male-specific lethal (MSL-2) protein, which is involved in X chromosome-dosage compensation in males, by inhibiting both splicing and translation of the *msl-2* transcript in females (65). Although the *Drosophila* dosage compensation mechanism is not conserved in mammals, there might be a conserved mechanism in *Drosophila* and mammals in the AS regulation by WTAP, which modulates histone modification. Comprehensive and comparative analysis of WTAP complex binding sites in RNA transcripts of *Drosophila* and mammalian cells will help to determine the molecular mechanism of underlying conserved AS regulation by the WTAP complexes.

In conclusion, we have identified and characterized AS events regulated by the WTAP complex *via* an inhibitory mechanism. AS leads to subsequent quantitative and qualitative differences in the transcriptome, thereby altering protein structure or abundance. Our data presented here provide examples of alternative splicing-dependent regulation of gene expression, which is a process likely to contribute to cell cycle regulation and histone modification.

## Experimental procedures

### Cell culture and gene silencing

HUVECs (CC-2519A, Lot# 234871) obtained from Lonza, were cultured in the EGM2 medium (culture medium supplemented with growth factors) (Lonza) and used within the first six passages. HeLa and HEK 293 cells were grown in Dulbecco’s modified Eagle’s medium supplemented with 10% fetal bovine serum. Dox-inducible HEK 293 stable cells expressing V5-SUV420H2 (protein coding sequence) were generated using the Flp-InTM T-RExTM core kit (Invitrogen) according to the manufacturer’s protocol. In total, 100 ng/ml doxycycline (BD Biosciences) was used for the induction. siRNAs against WTAP, Virilizer, BCLAF1, THRAP3, ZC3H13, CBLL1, RBM15, RBM15B, METTL3, and METTL14 were purchased from Ambion (s18431 and s18433 (referred as WTAPsi1), s24832, s18875, s19360, s23010, s36537, s224626, s266553, s32143, and s33679 respectively). siRNAs were transfected with lipofectamine 2000 RNAi MAX (Thermo Fisher Scientific) according to the manufacturer’s protocol.

### RNA isolation and RNA-seq analysis

The total RNA was isolated from the HUVEC treated with the siRNA of interest for 48 h using the Maxwell RNAeasy kit (Promega) and treated with DNaseI. PolyA+ mRNA was isolated from the total RNA and subjected to the preparation of the RNA-seq library. The resulting libraries were sequenced with Illumina NovaSeq 6000 (Illumina). The sequencing yielded an average of ∼39 million of 150 bp paired-end sequences per sample.

The RNA-sequencing reads were mapped to the human genome (GRCh38) using the aligner STAR (version 2.6.1c) with the additional parameters --outSJfilterReads Unique --outFilterType BySJout --outFilterMultimapNmax 10 --alignSJoverhangMin 6 --alignSJDBoverhangMin 3 --outFilterMismatchNoverLmax 0.1 --alignIntronMin 20 --alignIntronMax 1000000 --outSAMstrandField intronMotif --outFilterIntronMotifs RemoveNoncanonicalUnannotated.

### Differential gene expression analysis

The read count table was produced with the RSEM software (v1.3.1) using GENCODE gene annotation (release 33). The read counts were filtered with minimal counts per million as 0.5 in at least one library (iDEP.90, http://bioinformatics.sdstate.edu/idep/), then the differential expression analysis was performed using the R/Bioconductor package DESeq2. Genes were considered to be significantly and differentially expressed between control and KD of each component of the WTAP complex (*i.e.*, WTAP, VIRMA, ZC3H13, and CBLL1) (two replicates) based on the following criteria: FDR ≤0.01 and fold change ≥1.5. DEGs of WTAP KD sample treated with siRNA s18433 (one replicate) were analyzed using R/Bioconductor package TCC based on the criteria: FDR ≤0.01 and fold change ≥1.5. Overlaps of DEGs between WTAP KDs (using siRNA s18431 or siRNA s1843) were used as DEGs of WTAP KD for higher specificity. The gene ontology enrichment (GEO) analysis was performed using the GOrilla (http://cbl-gorilla.cs.technion.ac.il/). Hierarchical clustering was performed using normalized read counts. KD of other WTAP-interacting proteins (*i.e.*, BCLAF1/THRAP3, RBM15/RBM15B, METTL3, METTL14) (one replicate) were used for comparison.

### Differential isoform expression analysis

The raw transcript counts were extracted from the transcriptome alignment files using the RSEM software (https://deweylab.github.io/RSEM/) (v1.3.1). The DEIs were calculated using the R package EBSeq (https://git.bioconductor.org/packages/EBSeq) (v1.26.0). The additional parameters used were Qtrm = 0.5, QtrmCut = 10 in EBTest. Differential isoform usage between control and KD of each component of the WTAP complex (*i.e.*, WTAP, VIRMA, ZC3H13, and CBLL1) considered at FDR was below 0.01.

### Alternative splicing analysis

The splicing analyses were performed using VAST-TOOLS software (v2.2.2) (https://github.com/vastgroup/vast-tools), and the downstream analyses were performed using a Unix command-line toolkit matt (http://matt.crg.eu/). The command-line parameters for VAST-TOOLS to identify differential splicing events were as follows: vast-tools --min_dPSI 15 --paired --min_range 12. The comparison was performed between two replicates of control siRNA-treated samples *versus* siRNA-of-interest-treated samples (*i.e.*, WTAP, VIRMA, ZC3H13, and CBLL1), and between one replicate of siRNA of other WTAP-interacting proteins (*i.e.*, BCLAF1/THRAP3, RBM15/RBM15B, METTL3, METTL14) treated sample *versus* control siRNA-treated sample. All bioinformatics analyses were performed using the supercomputer SHIROKANE (Human Genome Center, University of Tokyo). The total number of events are output from VAST-TOOLS, which have been profiled (*i.e.*, that pass any applied filter in vast-tools) and are alternatively spliced in at least one of the compared samples (10 < PSI < 90). G4 RNA screener (http://scottgroup.med.usherbrooke.ca/G4RNA_screener/) was used for identification of potential G-quadruplex sequence in the regulated exon sequence and 3′ and 5′ss 100 nucleotides of the regulated introns, with the parameters of window size 60 and step size 10. Randomly sampled 1000 nondifferentially spliced exons and introns were used for comparison.

### Quantification of splicing

RT-PCR validation was conducted for several AS events randomly selected among cassette exons and intron retention (categorized as S or IR-S in vast-tool), which were commonly regulated by the four major components identified either in replicate 1 or replicate 2 (Table S3). The command-line parameters for VAST-TOOLS to identify commonly regulated splicing events were as follows: vast-tools --min_dPSI 15 --min_range 12. For RT-PCR validation, the total RNA was reverse-transcribed using the Superscript III with Oligo(dT) (Life Technologies). The PCR amplification was performed with Go Taq HS (Promega) for 30 to 35 cycles of 10 s at 98 °C, 30 s at 62 °C, and 30 s at 72 °C. After RT-PCR, the products were resolved by 4% TBE-PAGE. The PCR products were analyzed with Agilent DNA 1000 Kit (Agilent Technologies) run on a Bioanalyzer 2100 (Agilent Technologies). The primer sequences are included in Table S6.

### 3′RACE and nested qPCR

Total RNA was isolated using Maxwell RSC simplyRNA Cells Kit (Promega; cat#AS1340) and treated with DNase I. For the 3′ RACE, cDNA was synthesized by reverse transcription using 3′-Full RACE Core Set (TaKaRa; cat#6121) with oligo(dT)-containing adapter primer to poly (A) mRNA. Subsequently, a nested PCR method was used to amplify either *MSL1* or *WTAP* transcripts. The first PCR was performed with TaKaRa Ex-Taq HS (TaKaRa, cat#RR006A) (98 °C for 10 s, 62 °C for 30 s, and 72 °C for 1 min, ten cycles of amplification) using a *MSL1*-or *WTAP*-specific forward primer and the antisense adapter primer. For the secondary PCR, 0.5 μl of the PCR product from the initial amplification was used as template in a qPCR reaction (20 μl final volume) containing *MSL1*-or *WTAP*-specific forward and reverse primers located in the inner region of the first amplicons. TB Green Fast qPCR Mix (TaKaRa; cat#RR430S) was used for qPCR analyses performed with CFX96 Real-Time System (Biorad). The 3′ end of the resulting products was sequenced and confirmed using Sanger sequencing. Primer sequences used for 3′ RACE and nested Real-Time PCR are listed in Table S6.

### RNA coimmunoprecipitation (RIP) and quantitative RT-PCR

Prior to cell lysis, 75 μl of Dynabeads Protein G (Thermo Fisher Scientific; cat#10004D) was washed twice with 1 ml of RIPA buffer (50 mM Tris-HCl pH7.5, 1% NP40, 0.5% sodium deoxycholate, 0.05% SDS, 1 mM EDTA, 150 mM NaCl, protease inhibitor mixture, RNAsin), incubated with 10 μg of either anti-WTAP monoclonal antibody (H1122 (15)) or antiviral protein monoclonal antibody (anti-gp64, as negative control) in 200 μl of RIPA buffer at room temperature for 20 min while rotating, and then washed with 1 ml of RIPA buffer five times. HUVECs were grown in 150 mm dish, washed once with ice-cold PBS, and finally cross-linked with 1% Formaldehyde in PBS for 10 min. After neutralization with 0.2 M glycine, cells were washed once with ice-cold PBS, collected, resuspended in hypotonic buffer (10 mM HEPES (pH7.9) at 4 °C, 1.5 mM MgCl2, 10 mM KCK, 0.2 mM PMSF, protease inhibitor mixture, RNAsin), and incubated for 10 min on ice. Suspended cells were homogenized by eight passages through a 21-gauge needle, and the nuclei were collected using centrifugation for 10 min at 3000 rpm, resuspended in 1.5 ml RIPA buffer, sonicated (Sonifier 250, Branson; 4 min, 60% duty, output level 4), and then treated with 90 units of RQ1 DNAse (Promega, M6101) for 5 min at 37 °C. Following centrifugation for 10 min at 12,000*g* at 4 °C, the supernatant was frozen with liquid nitrogen and stored at −80 °C as the cell lysates. For immunoprecipitation, the antibody-conjugated Dynabeads Protein G was added to 400 μl of the cell lysates and incubated at 4 °C for overnight on a rotator. Beads were subsequently washed five times with 1 ml RIPA buffer and vortexed vigorously each time, then washed twice with 50 mM Tris-HCl (pH7.5), 50 mM NaCl, 2 mM EDTA, and incubated with 1000 units of RNase T1 for 5 min at 37 °C. After rinsing three times with RIPA buffer, Protein/RNA complexes were eluted and reverse cross-linked in 100 μl elution buffer (50 mM Tris-HCl (pH8.0), 10 mM EDTA, 1% SDS, 0.25 M NaCl supplemented with 1 mg/ml proteinase K) for 30 min at 55 °C. RNAs in the supernatant were extracted with 300 μl of TRIzol and precipitated from the aqueous phase with 200 μl of isopropanol and 0.5 μl of GlycoBlue (Thermo Fisher Scientific; cat#9515) used as a carrier. RNA pellets were washed with 75% ethanol, rehydrated in 8 μl nuclease-free water, and treated with RQ1 DNAse for 30 min at 37 °C, followed by addition of 1 μl stop solution and incubated for 10 min at 65 °C. Reverse transcription was conducted with SuperScript IV (Thermo Fisher Scientific; cat#18090010) using random hexamer primers following the manufacturer’s instructions. cDNA samples were analyzed by quantitative PCR (qPCR) using CFX96 Real-time detection System (BioRad) and TB Green Fast qPCR Mix. The primer combinations used for qPCR are listed in Table S6.

### Minigene assay

The SLC2A6 minigene was constructed by subcloning a PCR product of the SLC2A6 gene containing exon 7 to 25 nt downstream of exon 9 amplified from the human genomic DNA and cloned into the pCMV56 expression vector (Clontech). Next, 100 ng of the minigene plasmid was transfected to HUVECs plated in 6-well plates using Fugene HD (Promega). The cells were harvested after 24 h of incubation and used for RNA isolation and further semiquantitative RT-PCR analysis. The PCR amplification was performed using primers containing minigene-specific sequences (PT1 and PT2). The full sequence of the minigene is included in the Supplementary Materials.

### Cell cycle analysis

The cell cycle distribution was analyzed using flow cytometry. The HUVECs were transfected 48 h ago with the siRNA (s18433) and were subsequently rinsed with PBS, scraped and spun down for 5 min at 190*g*. The cell pellets were resuspended in PBS containing 0.2% Triton-X and subsequently incubated with 100 mg/ml RNase A followed by staining with propidium iodide (Sigma). The stained cells were analyzed using a FACSCalibur flow cytometer (BD Biosciences) for relative DNA content based on the red fluorescence levels. The percentage of cells in each of the cell cycle compartments was determined with ModFit LT software (Verity Software House).

### Antibodies

The monoclonal antibodies against CBLL1 (Y6018 and Y6037, 430–491 amino acids were used as an immunogen) and VIRMA (Y1639, 1421–1480 amino acids were used as an immunogen) were generated using a baculoviral display system, as described previously (66). Polyclonal anti-human WTAP antibodies raised in rabbits were used for immunoblotting (16). The following antibodies were used for immunoblot analysis: ZC3H13 (ab70802, Abcam), BCLAF1 (A300-608A, Bethyl), THRAP3 (A300-956A, Bethyl), CBLL1 (ARP39623_T100, Aviva Systems Biology), RBM15 (ab70549, Abcam), RBM15B(1C2C11, Proteintech), METTL3 (D2I6O, Cell Signaling Technology), METTL14(ab220030, Abcam), H4K20me1 (ab9051, Abcam), H4K20me2 (ab9052, Abcam), H4K20me3 (ab9053, Abcam), beta-Actin (A5441, Sigma), V5(MA5-15253, Invitrogen) and FLAG(F7425, Sigma Aldrich).

### Western blotting

The HUVEC, HEK293, and HeLa cells were lysed in lysis buffer (20 mm HEPES, pH 7.9, at 4 °C, 10% glycerol, 250 mM KCl, 0.2 mm EDTA, 0.1% NP-40, 0.2 mm PMSF) with protease inhibitors (Roche) and 50 U Benzonase (Novagen) for 30 min on ice. The lysate was centrifuged (12,000*g*; 15 min), and the supernatant was collected as whole cell lysates. Total protein was quantified using the PierceTM BCA Protein Assay Kit (Thermo Fisher Scientific) following the manufacturer’s instructions. The proteins were resolved by SDS-PAGE and transferred onto a nitrocellulose membrane for immunoblotting following standard procedures. Blocking was conducted with BlockAce (Yukijirushi) for 1 h at room temperature, and membranes were incubated overnight at 4 °C with a primary antibody. Incubation with the secondary antibody goat anti-rabbit IgG-HRP (Santa Cruz Biotechnology, sc-2004) or anti-mouse IgG-HRP was conducted for 1 h at room temperature. For chemiluminescent detection, the ECL Prime Western Blotting Detection Reagent (GE Healthcare Life Sciences) was used following the manufacturer’s instructions. The monoclonal anti-alpha-tubulin antibody was used as the loading control. For histone detection, the cell pellets were lysed in SDS buffer and subjected to western blotting.

### Immunopurification of CBLL1-interacting proteins

HUVECs were grown to 75 to 80% confluence in a 150 mm dish, harvested, suspended in 1 ml lysis buffer (20 mm HEPES, pH 7.9, at 4 °C, 10% glycerol, 250 mM KCl, 0.2 mm EDTA, 0.1% NP-40, 0.2 mm PMSF) with protease inhibitors (Roche) and 50U Benzonase (Novagen), and incubated on ice for 30 min. After centrifugation for 30 min at 12,000*g* at 4 °C, the supernatant was frozen with liquid nitrogen and stored at −80 °C as the whole cell lysate. Immunopurification was performed as described previously (15). Briefly, 1.2 mg of filtered whole cell lysates using 0.22 μm filter units was incubated for 4 h at 4 °C with 2 μg antibody against CBLL1 cross-linked to Dynabeads Protein G (Thermo Fisher Scientific) with dimethyl pimelimidate. Magnet beads were washed three times with 0.5 m KCl-HEGN (20 mm HEPES, pH 7.9, at 4 °C, 0.5 M KCl, 0.1 mM EDTA, 10% glycerol, 0.1% Nonidet P-40,) and once with 0.1 M KCl-HEGN (20 mm HEPES, pH 7.9, at 4 °C, 0.1 m KCl, 0.1 mM EDTA, 10% glycerol, 0.1% Nonidet P-40) at 4 °C. The affinity-purified proteins were eluted by 0.05% RapiGest (Waters) in 50 mm NH4HCO_3_ buffer for 5 min at 95 °C. The eluent was concentrated by 10% ice-cold TCA, washed with ice-cold acetone, and dried.

### Liquid chromatography–tandem mass spectrometry (LC/MS/MS)

The immunopurified (IP) samples underwent in-solution trypsin digestion followed by LC/MS/MS using the LTQ XL mass spectrometers (Thermo Fisher Scientific). The mass spectrometer was operated in a data-dependent acquisition mode, in which the MS acquisition with a mass range of *m/z* 450 to 1800 was automatically switched to MS/MS acquisition under the automated control of Xcalibur software. The top three precursor ions were selected using an MS scan and for the subsequent MS/MS scans by ion trap in the normal/centroid mode, using the automated gain control (AGC) mode with AGC values of 3.00 × 10^4^ and 1.00 × 10^4^ for full MS and MS/MS, respectively. We also employed a dynamic exclusion capability that allowed sequential acquisition of the MS/MS of abundant ions in the order of their intensities with an exclusion duration of 35 s and exclusion mass widths of −1 and +2 Da. The trapping time was 200 ms with the auto gain control on. Tandem mass (MS/MS) spectra were extracted using Proteome Discoverer version 2.2. All MS/MS samples were analyzed using Mascot (Matrix Science; version 2.6.2). Mascot was set up to search against the human SwissProt database (selected for Homo Sapiens, 2020.05 version, 20,386 entries) assuming digestion with trypsin. The fragment and parent ion mass tolerances were 2.4 Da and 1.2 Da, respectively. A maximum of three missed cleavages were allowed. Carbomidomethyl of cysteine was specified in Mascot as a fixed modification. Gln->pyro-Glu of the n-terminus, oxidation of methionine, acetyl of the n-terminus, and phospho of serine, threonine, and tyrosine were specified in Mascot as variable modifications. Data analysis was performed using Scaffold software (version 4.10; Proteome Software Inc). Peptide identification was allowed at FDR of <1.0%. Protein identification was allowed at FDR of <1.0% and contained at least two identified peptides.

### Statistics

All experiments were performed a minimum of three times. Data points represent the mean ± SD calculated from multiple independent experiments. Statistically significant differences were calculated using either the unpaired *t* test or analysis of variance and Tukey–Kramer multiple comparisons tests using R version 3.6.2 (https://www.R-project.org/). The *p*-values less than 0.05 were considered significant unless described otherwise.

## Data availability

RNA-sequencing data have been deposited in GEO (https://www.ncbi.nlm.nih.gov/geo/) under accession number GSE 167067. The mass spectrometry data have been deposited to the ProteomeXchange Consortium *via* the Jpost partner repository (67) with the data set identifier PXD027423.

## Supporting information

This article contains supporting information (15).

## Conflict of interest

The authors declare that they have no conflicts of interest with the contents of this article.
