# Supplementary material for: Wilms’ tumor 1-associating protein complex regulates alternative splicing and polyadenylation at potential G-quadruplex-forming splice site sequences
Source: J Biol Chem. 2021 Sep 25;297(5):101248. doi: 10.1016/j.jbc.2021.101248 (PMC8605363; doi:10.1016/j.jbc.2021.101248)
Supplement: Supplemental Figures S1–S6 and Table S6 [file mmc2.pdf]

SUPPORTING INFORMATION

Wilms' Tumor 1-Associating Protein complex regulates alternative splicing and polyadenylation at potential G-quadruplex-forming splice site sequences

Keiko Horiuchi[1]*, Takeshi Kawamura[2], and Takao Hamakubo[1]*

[1] Department of Protein-Protein Interaction Research, Institute for Advanced Medical Sciences, Nippon Medical School, Tokyo, 113-0011, Japan
[2] Laboratory for Systems Biology and Medicine, Research Center for Advanced Science and Technology, The University of Tokyo, 4-6-1, Komaba, Meguro-ku, Tokyo 153-8904, Japan

SUPPLEMENTARY TABLES

Supplementary Table S1. Gene expression analysis of RNA-seq data, shown as TPM (transcripts per million) calculated by RSEM software across all treatment conditions.

Supplementary Table S2. Gene isoform expression analysis of RNA-seq data, shown as TPM (transcripts per million) calculated by RSEM software across all treatment conditions.

Supplementary Table S3. Splicing analysis of RNA-Seq data showing PSI (percent spliced in) or PIR (percent intron retention) values for all treatment conditions. Results are in hg38.

Supplementary Table S4. List of predicted rG4 sequences linked to AS change by KD of WTAP complex.

Supplementary Table S5. List of isolated proteins from immunopricipitates using anti-CBLL1 from HUVECs whole cell lysates. Anti-viral gp64 antibody (mouse monoclonal) was used as negative control. Published WTAP-interacting proteins are also listed as reference[15].

Supplementary Table S6. List of oligonucleotide primers used in the study.

Supplementary References

15. Horiuchi, K. et al. Identification of Wilms' tumor 1-associating protein complex and its role in alternative splicing and the cell cycle. J Biol Chem 288, 33292-302 (2013).

Supplementary Table S6 Primers used in the study

for RT-PCR

GENE	Forward	Reverse-1	Reverse-2
ABCD4	GACAGAGGAAGTGGAGAGCGA	TCGATCTTCGCTGTCAGTCCT	
PTAR1	GCGGCATATTTTCTACCTTCAGC	CCTGTGCTCCATTTCTAGGCC	
CCNT2	ACCCACTGTTCTGAGGAGTCC	GTCCATATGCTGGCTGCTTGT	
YTHDC1	GGTTGGTGAACAGTTGCTCCA	AGCCCATTCGTAACACACACC	
IRF7	ATAACACCTGACCGCCACCTA	GAAGTGCTTCCAGGGCACG	
MSL1	AGATTTGCCGTACCTTTCCA	ATCCAGCTCCAGTTTTGCAT	GCCAAAGGCATTTTCACACT
WTAP	GCGACTAGCAACCAAGGAAC	CATTCGACACTTCGCCATTA	TGAGTCTTGGTGTGGAAACG
EPB49	GCCTGACACGCTGTCCTCTC	CTCGATGTCCAGGATGGCCTT	
EGFL7	TAGGGTGTGTGCTGTCCGG	CTGACTGGCAAGTGTCACCC	
EXD3	GAGGTGACCTCCTTGAGCCT	TCGGTCCAGTTCTCCTGTGAC	
SUV420H2	CACGAGAACTGTGCGAGAAC	CTTTTTCCAAGCACGAGTGG	
HDAC7	TCCTCCCCAAGTAGTAGCAGC	GAGGGGTCCAGGAGGAGAATG	
SLC2A6	CAAGGACGACGCAGCCATC	GCAGGAAGGACTTGGTGAGGA	

for RIP-qPCR

GENE	Forward	Reverse
SLC2A6	GCTCCTGTCCGTGCTGAT	CTGCCTGCAGGGTGCTTAC
GAPDH	CCCCTTCATACCCTCACGTA	GACAAGCTTCCCGTTCTCAG

for mini-gene

PT1	GTCGACGACACTTGCTCAAC
PT2	AAGCTTGCATCGAATCAGTAG

for 3'RACE

3sites Adaptor primer	CTGATCTAGAGGTACCGGATCC
MSL1-forward primer	AGATTTGCCGTACCTTTCCA
WTAP-forward primer	GGGTTTATTTGCTGAGAACCA

for nested qPCR (for alternatively polyadenylated isoform)

GENE	Forward	Reverse
MSL1	AGGGGCCAATGGTAATTTTT	TGGTCTTTGGCAGATTCCTC
WTAP	TTTATGAAATCCCCGTTCCA	GGCCCAAATACAATGAAGGA

for isoform specific qPCR (for exon skip/inclusion)

GENE	Forward	Reverse	
MSL1	AGATTTGCCGTACCTTTCCA	GGTCCCTCCAAGAAGGAATT	exon4 skip
MSL1	AGATTTGCCGTACCTTTCCA	TCTCCTGCAACACTTGATGG	exon4 inclusion
WTAP	ACTGGAACAAGCCCAAAATG	TGCCTTCCAAGCTCTTGATT	intron5 splicing

SUPPLEMENTARY METHODS

The full sequence of the *SLC2A6* minigene, which corresponds to HsaEX0059304 in vastdb (https://vastdb.crg.eu/wiki/Main_Page).

Capital letters denote exon sequences. The predicted rG4 regions are marked in green or yellow.

CCCCCCAAGGACGACGCAGCCATCGTTGGGGCCGTGCGGCTCCTGTCCGTGCTGATCGCCGCC
CTCACCATGGACCTCGCAGGCCGCAAGGTGCTGCTCTTCGTCTCAGgtaagcaccctgcaggcagccctcct
gccccactcctccgcatgggtcagggctgggctgggtgtgcaggcctgcctcggggtggggctgtgtggctgggaactgcagagcacctgcc
tccctagcatcggcagggccctggcccaggccagtggtccagaaaggtccgctggggctccgagtggacacactggctgtgacactccacg
gtgggagcctaacacgcggctggagacagagcttgctgccctgctgggggtctctagtcccagacacgagctggaccacgcaagtatcaag
gggctctgcagaaggagctctgagagaaacagctggctcagagggtcctggccccacgaggccacctgtctgtccacagCGGCCATC
ATGTTTGCTGCCAACCTGACTCTGGGGCTGTACATCCACTTTGGCCCCAGGCCTCTGAGCCCCA
ACAGCACTGCGGGCCTGGAAAGCGAGTCCTGGGGGGACTTGGCGCAGCCCCTGGCAGCACCC
GCTGGCTACCTCACCCTGGTGCCCCTGCTGGCCACCATGCTCTTCATCATGGgtaggtgtggtggtggct
cagaggggcaggctgtcttgggtgttaagggatgggtgatgtgtctggggtggctggagaggggggtctccagcagctcagcggagacaga
cacagccgcctccagtcaccccacagggcctgaacctgcctcctcctccgagcagGCTACGCCGTGGGCTGGGGTCCCAT
CACCTGGCTGCTCATGTCTGAGGTCCTGCCCCTGCGTGCCCGTGGCGTGGCCTCAGGGCTCTG
CGTGCTGGCCAGCTGGCTCACCGCCTTCGTCCTCACCAAGTCCTTCCTGCCAGTGGTGgtgagtgtt
cagccccaggccccag

rG4 mutation in downstream intron:

WT : ggggcaggctgtcttgggtgttaagggatgggtgatgtgtctggggtggctggagaggggggg

mt1 : gCCgcaggctgtcttgAgtgttaagAgatCCCtgatgtgtctATggtggctggagaggCCgg

rG4 mutation in upstream intron:

WT : gggctgggctgggtgtgcaggcctgcctcggggtggggctgtgtggctggg

mt2: gTgctgAgctgggtgtgcaggcctgcctcgCgCtggATctgtgtggctgCg

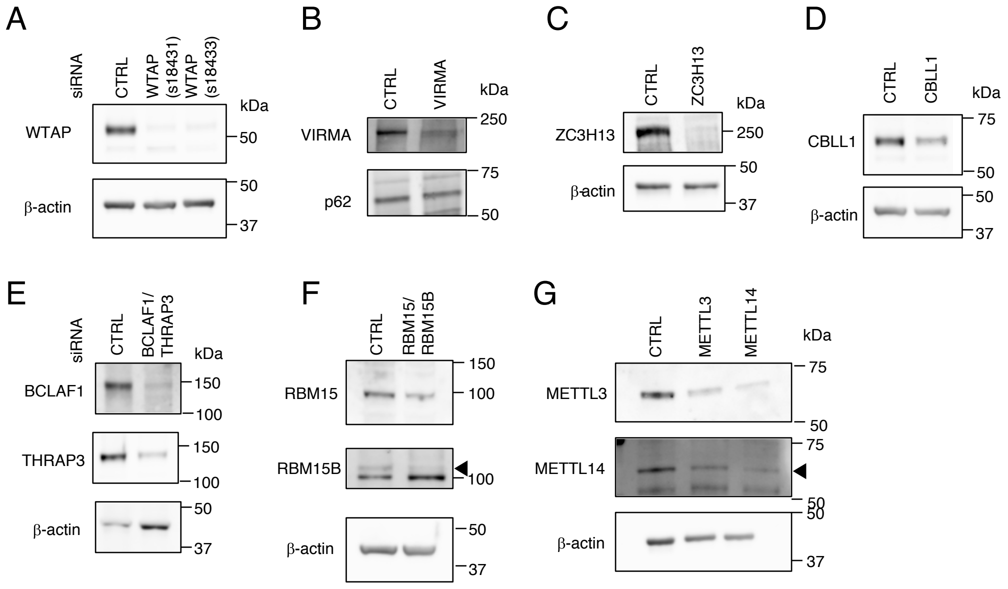

Supplementary Figure S1. KD efficiency of WTAP-interacting proteins by siRNA. Efficient reduction of WTAP and its interacting proteins (A) WTAP, (B) VIRMA, (C) ZC3H13, (D) CBLL1, (E) BCLAF1 and THRAP3, (F) RBM15 and RBM15B, and (G) METTL3 and METTL14, by siRNA was confirmed by western blot using whole-cell extracts from HUVECs transfected with siRNA of WTAP-interacting proteins or control. Total proteins were extracted 48 h after transfection. For the detection of VIRMA, the nuclear extract was used to detect the endogenous protein. Beta-actin or nucleoporin (p62, for VIRMA) was used as a loading control.

Supplementary Figure S2

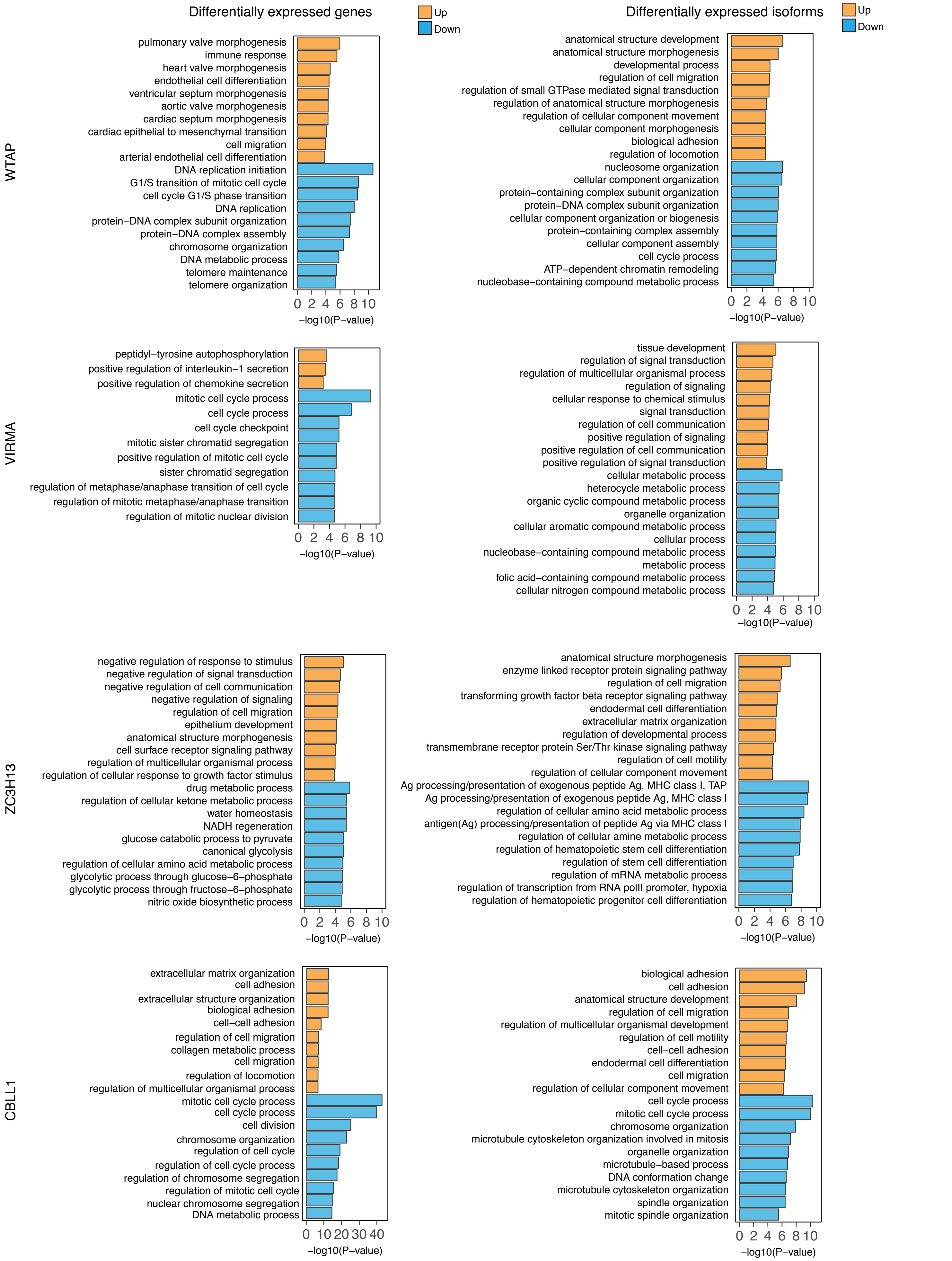

Supplementary Figure S2. Gene ontology analysis of the differentially expressed genes (DEG) and isoforms (DEI). GO enrichment analysis of either increased (up) or decreased (down) DEG and DEI by knockdown of each of WTAP core factors, WTAP, VIRMA, ZC3H13, and CBLL1 was performed using Gorilla (http://cbl-gorilla.cs.technion.ac.il/). FDR ≤ 0.01 and fold change ≥ 1.5. Enrichment of the functional categories in the biological process is shown according to the p-value.

Supplementary Figure S3

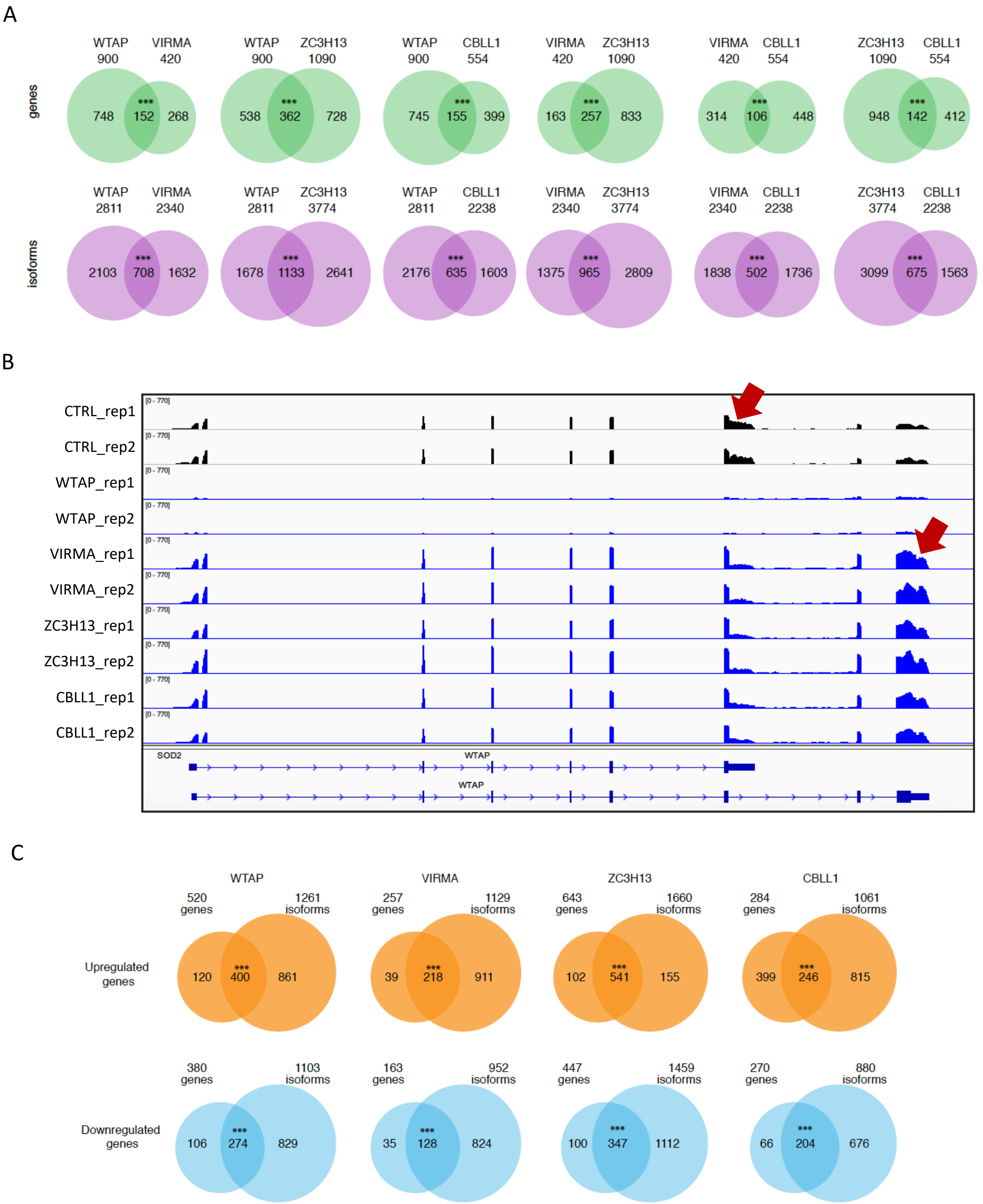

Supplementary Figure S3. Overlap of changes between gene and isoform expressions.
(A) Venn diagrams showing the overlaps of genes identified as DEG or DEI between two groups. (B) WTAP complex autoregulates splicing/polyadenylation of the WTAP pre-mRNA. RNA sequencing depth-of-coverage profiles of the WTAP gene were visualized using IGV. Arrows indicate the difference of the read depth which corresponds to each alternative isoform. (C) Venn diagrams showing the overlaps of either increased (upper panel) or decreased (lower panel) DEG and DEIs in WTAP major component KD samples. Statistical significance of overlap of two groups was assessed using Fisher's exact test ***P < 0.0001.

Supplementary Figure S4

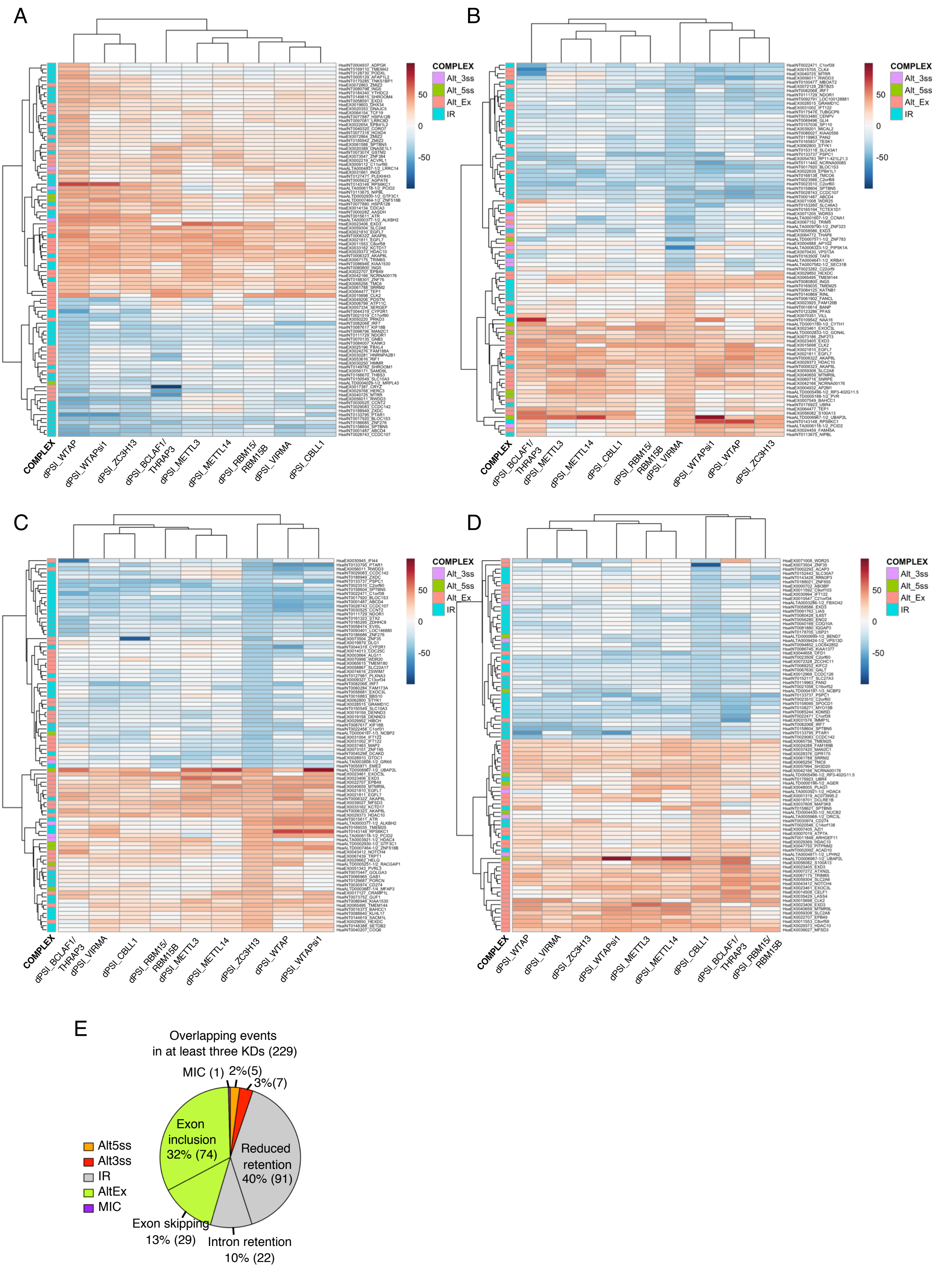

Supplementary Figure S4. Clustering analysis of affected AS evets by KD of the WTAP complex . Heatmap and hierarchical clustering of top100 regulated AS events by KD of each of the major components, (A)WTAP, (B)VIRMA, (C)ZC3H13, and (D)CBLL1. (E) Distribution of affected AS events with dPSI or dPIR ≥ 15 in KD of at least three of the major components.

Supplementary Figure S5

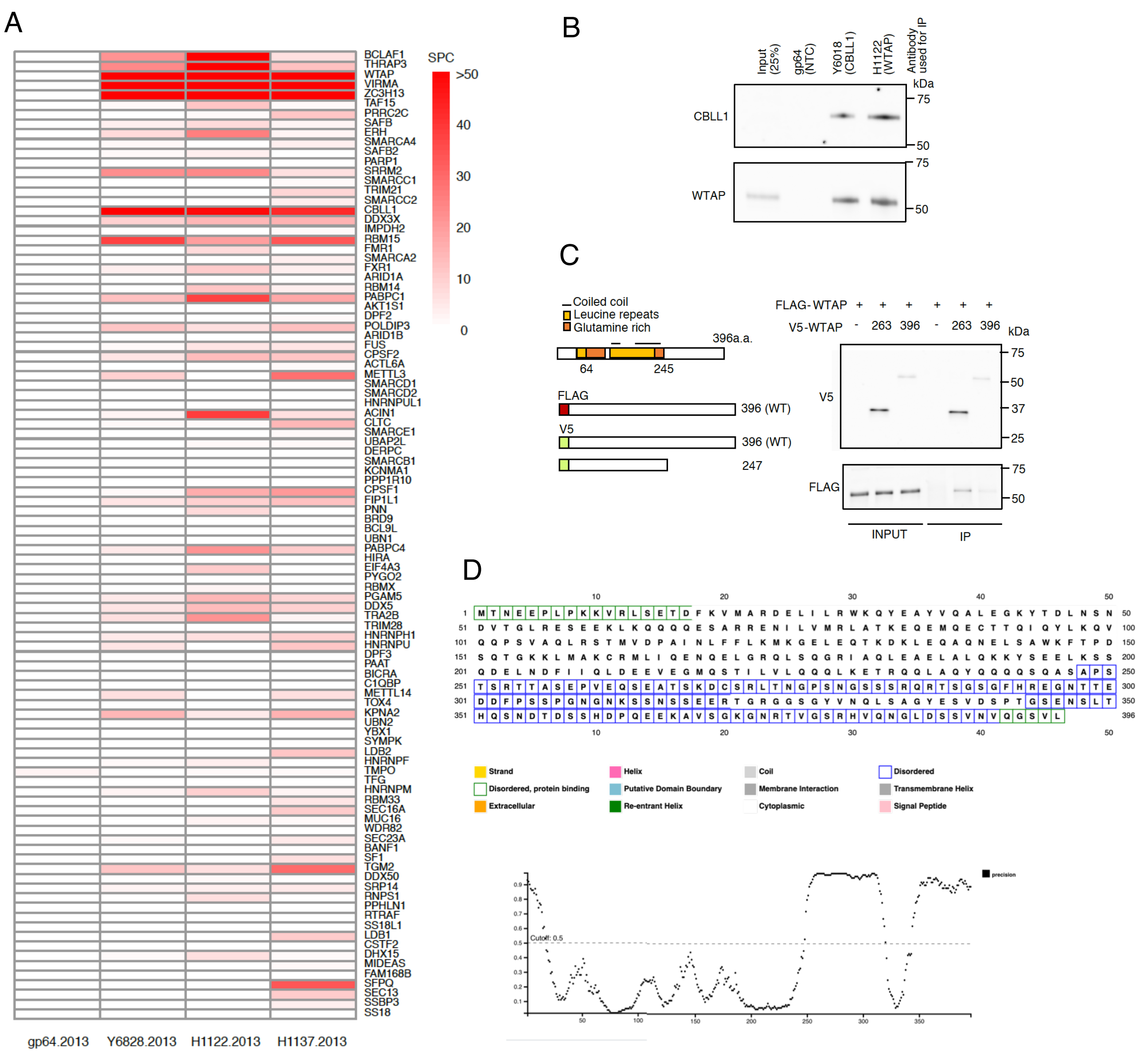

Supplementary Figure S5. Immunoprecipitation (IP) and Immunoblotting analysis shows stable interaction between WTAP and CBLL1. (A) The proteomic profile of WTAP-interacting proteins (published data[1]) for comparison with that of CBLL1. The top 100 identified proteins were ordered by total spectrum counts from Y6018 and Y6037 immunoprecipitates. (B) IP and Immunoblotting demonstrating that majority (~70%) of WTAP was recovered by CBLL1 IP using the Y6018 antibody. Nearly all CBLL1 protein was recovered by WTAP IP with the H1122 antibody. The anti-gp64 (mouse monoclonal, K7124) antibody was used as a negative control antibody for IP. (C) (Left) Schematic construct of V5- and FLAG-tagged WTAP and C-terminal deletion mutant. (Right) Western blot of Co-IP using whole cell lysates from HEK293 cells transfected together with full length FLAG-tagged WTAP and full length or C-terminal truncated V5-tagged WTAP. (D) Amino acid sequences of WTAP and predicted disordered region analyzed by DISOPRED3.

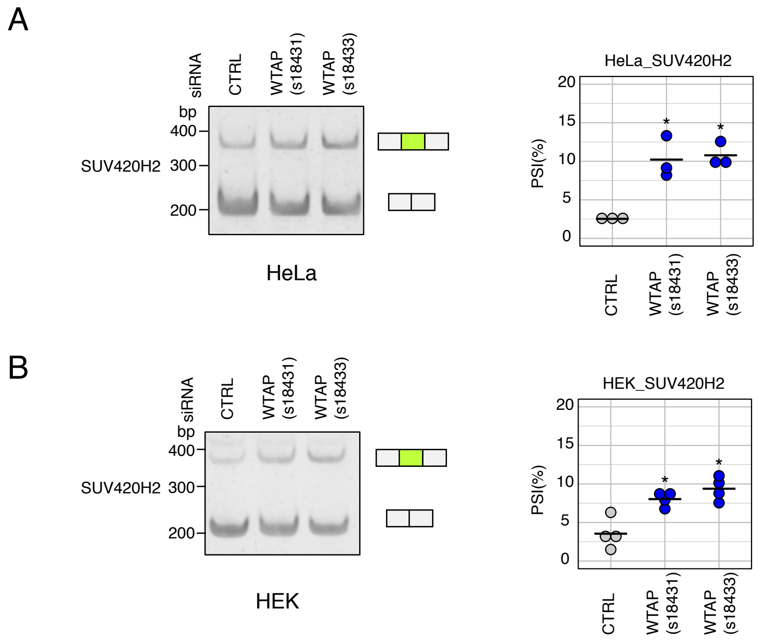

Supplementary Figure S6. RT-PCR validation of SUV420H2 AS events. KD of WTAP leads to an increase of exon 3 inclusion of SUV420H2 gene also in (A) HeLa and (B) HEK293 cells. Representative image of PCR products and quantification of the percentage of exon 3 inclusion for three independent biological replicates. The values are the average ± SD (error bars), *p < 0.01 versus control siRNA treated cells (t-test).
